# Risk of COVID‐19 Hospitalization and Protection Associated With mRNA Vaccination Among US Adults With Psychiatric Disorders

**DOI:** 10.1111/irv.13269

**Published:** 2024-03-17

**Authors:** Matthew E. Levy, Duck‐Hye Yang, Margaret M. Dunne, Kathleen Miley, Stephanie A. Irving, Shaun J. Grannis, Zachary A. Weber, Eric P. Griggs, Talia L. Spark, Elizabeth Bassett, Peter J. Embi, Manjusha Gaglani, Karthik Natarajan, Nimish R. Valvi, Toan C. Ong, Allison L. Naleway, Edward Stenehjem, Nicola P. Klein, Ruth Link‐Gelles, Malini B. DeSilva, Anupam B. Kharbanda, Chandni Raiyani, Maura A. Beaton, Brian E. Dixon, Suchitra Rao, Kristin Dascomb, Palak Patel, Mufaddal Mamawala, Jungmi Han, William F. Fadel, Michelle A. Barron, Nancy Grisel, Monica Dickerson, I‐Chia Liao, Julie Arndorfer, Morgan Najdowski, Kempapura Murthy, Caitlin Ray, Mark W. Tenforde, Sarah W. Ball

**Affiliations:** ^1^ Westat Rockville Maryland USA; ^2^ Helix San Mateo California USA; ^3^ HealthPartners Institute Minneapolis Minnesota USA; ^4^ Kaiser Permanente Center for Health Research Portland Oregon USA; ^5^ Center for Biomedical Informatics Regenstrief Institute Indianapolis Indiana USA; ^6^ School of Medicine Indiana University Indianapolis Indiana USA; ^7^ Coronavirus and Other Respiratory Viruses Division, National Center for Immunization and Respiratory Diseases Centers for Disease Control and Prevention Atlanta Georgia USA; ^8^ Vanderbilt University Medical Center Nashville Tennessee USA; ^9^ Baylor Scott & White Health Temple Texas USA; ^10^ Texas A&M University College of Medicine Temple Texas USA; ^11^ Department of Biomedical Informatics Columbia University Irving Medical Center New York New York USA; ^12^ New York Presbyterian Hospital New York New York USA; ^13^ School of Medicine University of Colorado Anschutz Medical Campus Aurora Colorado USA; ^14^ Division of Infectious Diseases and Clinical Epidemiology Intermountain Healthcare Salt Lake City Utah USA; ^15^ Kaiser Permanente Vaccine Study Center Kaiser Permanente Northern California Division of Research Oakland California USA; ^16^ Children's Minnesota Minneapolis Minnesota USA; ^17^ Fairbanks School of Public Health Indiana University Indianapolis Indiana USA; ^18^ Influenza Division, National Center for Immunization and Respiratory Diseases Centers for Disease Control and Prevention Atlanta Georgia USA

**Keywords:** anxiety disorders, COVID‐19, electronic health records, epidemiology, mental disorders, mood disorders, psychotic disorders, vaccination

## Abstract

**Background:**

Although psychiatric disorders have been associated with reduced immune responses to other vaccines, it remains unknown whether they influence COVID‐19 vaccine effectiveness (VE). This study evaluated risk of COVID‐19 hospitalization and estimated mRNA VE stratified by psychiatric disorder status.

**Methods:**

In a retrospective cohort analysis of the VISION Network in four US states, the rate of laboratory‐confirmed COVID‐19‐associated hospitalization between December 2021 and August 2022 was compared across psychiatric diagnoses and by monovalent mRNA COVID‐19 vaccination status using Cox proportional hazards regression.

**Results:**

Among 2,436,999 adults, 22.1% had ≥1 psychiatric disorder. The incidence of COVID‐19‐associated hospitalization was higher among patients with any versus no psychiatric disorder (394 vs. 156 per 100,000 person‐years, *p* < 0.001). Any psychiatric disorder (adjusted hazard ratio [aHR], 1.27; 95% CI, 1.18–1.37) and mood (aHR, 1.25; 95% CI, 1.15–1.36), anxiety (aHR, 1.33, 95% CI, 1.22–1.45), and psychotic (aHR, 1.41; 95% CI, 1.14–1.74) disorders were each significant independent predictors of hospitalization. Among patients with any psychiatric disorder, aHRs for the association between vaccination and hospitalization were 0.35 (95% CI, 0.25–0.49) after a recent second dose, 0.08 (95% CI, 0.06–0.11) after a recent third dose, and 0.33 (95% CI, 0.17–0.66) after a recent fourth dose, compared to unvaccinated patients. Corresponding VE estimates were 65%, 92%, and 67%, respectively, and were similar among patients with no psychiatric disorder (68%, 92%, and 79%).

**Conclusion:**

Psychiatric disorders were associated with increased risk of COVID‐19‐associated hospitalization. However, mRNA vaccination provided similar protection regardless of psychiatric disorder status, highlighting its benefit for individuals with psychiatric disorders.

## 1 Introduction

Largely prior to the availability of COVID‐19 vaccines, psychiatric disorders and especially severe psychiatric disorders such as bipolar and psychotic disorders have been identified as risk factors for severe COVID‐19, COVID‐19‐associated hospitalization, and mortality [1, 2, 3, 4, 5]. Possible reasons include impaired immune function, chronic inflammation, comorbid medical conditions, behavioral risk factors (e.g., smoking, substance abuse), and barriers to accessing healthcare services [6, 7, 8, 9, 10].

Little is known regarding risk of COVID‐19 and associated outcomes among persons with psychiatric disorders who are vaccinated. In a US study conducted before Omicron (B.1.1.529) variant predominance, numerous psychiatric disorder diagnoses were associated with increased risk of SARS‐CoV‐2 infection among adults vaccinated with a COVID‐19 primary series [11]. Another study conducted in Taiwan during Omicron predominance identified psychiatric diagnoses and being unvaccinated as risk factors for COVID‐19 hospitalization but did not evaluate the effects of booster doses [8]. The extent to which this increased risk persists during Omicron variant predominance and after receipt of booster dose(s) remains unknown.

Further, although previous research has demonstrated that psychiatric symptoms (e.g., depressive symptoms, stress) can contribute to a reduced immune response to other vaccines including influenza, measles, hepatitis B, and varicella zoster vaccines [12, 13], little is known about whether psychiatric disorders influence COVID‐19 vaccine effectiveness (VE). In one study, depression was associated with lower antibody positivity after a primary vaccine series [14]. Data are lacking from real‐world studies on COVID‐19 VE in persons with psychiatric disorders.

In this study among adults during a period of Omicron variant predominance, our objectives were to (1) estimate the association between psychiatric disorders and risk of COVID‐19‐associated hospitalization overall and by COVID‐19 vaccination status, age group, and type of psychiatric disorder and (2) estimate and compare VE of two, three, and four mRNA vaccine doses against COVID‐19‐associated hospitalization among persons with and without psychiatric disorders.

## 2 Methods

### 2.1 Design and Setting

Longitudinal data from electronic health records (EHRs) were collected from four health systems and research centers in Indiana, Oregon, Texas, and Utah that partner with the US Centers for Disease Control and Prevention (CDC) and Westat® as part of the VISION Network [15, 16]. Reflecting a retrospective cohort study design, partners established inclusion criteria based on health insurance membership, medical utilization, and other criteria to ensure complete data on medical encounters, SARS‐CoV‐2 laboratory testing, and COVID‐19 vaccination for eligible patients (aged ≥18 years) (eTable 1 in the Supporting Information section). To maximize the likelihood that cohort participants were active patients with diagnoses available, all network partners required, at a minimum, that patients have ≥1 International Classification of Diseases (ICD) code (any) from a medical encounter during a 1‐year historical period between August 26, 2020, and August 25, 2021. Patients were excluded from this analysis if they had ≥1 ICD code for an immunocompromising condition [17].

Patients contributed follow‐up time from December 16 to 26, 2021 (i.e., the date when the SARS‐CoV‐2 Omicron variant first accounted for ≥50% of sequenced specimens in each network partner's state) [18], through August 30, 2022 (i.e., day before the US Food and Drug Administration authorized COVID‐19 bivalent vaccine boosters in adults) [19]. Patients were followed until COVID‐19‐associated hospitalization or a censoring event: an exclusionary COVID‐19 vaccine dose, departure from the health network, death, or August 30, 2022, whichever was earliest. Exclusionary doses included non‐mRNA vaccine doses, third or fourth mRNA doses before they were recommended, or doses with shorter intervals than recommended (i.e., <5 months between second and third or <4 months between third and fourth). Such doses resulted in either exclusion or censoring (upon receipt) depending on whether they occurred before (exclusion) or during (censoring) the follow‐up period. Among adults not moderately or severely immunocompromised, only those aged ≥50 years were eligible to receive a fourth dose during the study period, starting on March 29, 2022. Patients with known prior SARS‐CoV‐2 infection were not excluded, and any infection was not considered a censoring event because infection history was under‐ascertained, and this analysis focused on hospitalized cases as a severe outcome, including those resulting from reinfection.

### 2.2 COVID‐19‐Associated Hospitalization

The outcome was incident laboratory‐confirmed COVID‐19‐associated hospitalization, defined as a hospitalization with ≥1 COVID‐19‐like illness discharge diagnosis and a positive molecular or antigen SARS‐CoV‐2 test result documented in EHRs within 14 days before through <72 h after admission. COVID‐19‐like illness diagnoses included ICD codes for acute respiratory illness (e.g., respiratory failure, pneumonia) and related signs or symptoms (e.g., cough, fever, dyspnea) (eTable 2 in the Supporting Information section).

### 2.3 Psychiatric Disorders

Psychiatric disorders were defined using ICD codes documented at least once in inpatient or outpatient clinical settings during a historical lookback period. To maximize validity of diagnosis‐based measures, the lookback duration was a minimum of 1 year, with the specific duration determined by each partner based on their knowledge of their EHR‐based data source (eTable 1 in the Supporting Information section). Disorders included mood, anxiety, trauma‐/stressor‐related, psychotic, somatoform, attention‐deficit hyperactivity, eating, personality, and dissociative or conversion disorders (eTable 3 in the Supporting Information section). Patients could have multiple disorder types. Each ICD code corresponded with only one disorder type. Patients with no ICD codes for psychiatric disorders were classified as not having a psychiatric disorder. Data on self‐reported psychiatric symptoms or on psychiatric treatments were not available.

### 2.4 COVID‐19 Vaccination Status

COVID‐19 vaccination was ascertained through EHRs and linkages with state immunization information systems. Only mRNA vaccines (BNT162b2 [Pfizer‐BioNTech] or mRNA‐1273 [Moderna]) were considered. Vaccination status was defined as a time‐varying variable based on the number and timing of doses that had been received prior to each date. Patients were classified as unvaccinated (no COVID‐19 vaccine doses), vaccinated with two doses (second dose 14–149 or ≥150 days earlier), vaccinated with three doses (third dose 7–119 or ≥120 days earlier), and among patients aged ≥50 years, vaccinated with four doses (fourth dose 7–59 or ≥60 days earlier) [16]. We excluded person‐time with only one dose, the second dose 1–13 days earlier, or third or fourth dose 1–6 days earlier. Patients in one of those categories at the start of the period could enter the analytic cohort later if they became eligible based on a new vaccination status. Patients who received the Ad26.COV2.S (Janssen/Johnson & Johnson) vaccine were excluded because, among patients with a psychiatric disorder, the number of Ad26.COV2.S recipients was 4% the sample size of mRNA‐only vaccine recipients, limiting ability to calculate precise estimates when stratifying by number and timing of doses.

### 2.5 Statistical Analysis

#### 2.5.1 Descriptive Characteristics

Baseline characteristics were summarized among all patients and stratified by whether ≥1 psychiatric disorder was diagnosed using frequencies and proportions or medians and interquartile ranges (IQRs). We evaluated differences by psychiatric disorder status using standardized mean differences (SMDs).

#### 2.5.2 Association Between Psychiatric Disorders and COVID‐19‐Associated Hospitalization

We calculated unadjusted incidence rates and plotted Kaplan–Meier curves to compare risk of COVID‐19‐associated hospitalization between patients with any (vs. no) psychiatric disorder and by disorder type. Hazard ratios (HRs) and 95% confidence intervals (CIs) comparing time to hospitalization by psychiatric disorder status were calculated using multivariable Cox proportional hazards regression with a calendar time scale. Models adjusted for potential confounders including site, age (smoothed using natural cubic splines with four knots), sex, race and ethnicity (as documented in EHR), Medicaid coverage (proxy measure of socioeconomic status), time‐varying COVID‐19 vaccination status, ≥1 underlying respiratory condition diagnosis, ≥1 underlying non‐respiratory condition diagnosis (non‐psychiatric), total number of known respiratory and non‐respiratory underlying medical conditions (square‐root transformed), and number of SARS‐CoV‐2 test records in the EHR before the study period (0, 1, 2–4, ≥5). Variance inflation factors for all covariates were confirmed to be <5. Analyses were stratified by age group (18–49, 50–64, and ≥65 years) and COVID‐19 vaccination status. Interaction terms for age group or vaccination status with psychiatric disorder were evaluated. *P* < 0.05 indicated statistical significance.

#### 2.5.3 mRNA COVID‐19 VE by Psychiatric Disorder Status

We similarly estimated the association between time‐varying vaccination status and time to COVID‐19‐associated hospitalization using multivariable Cox proportional hazards regression. Patients with and without a psychiatric disorder were analyzed separately. Each two‐dose, three‐dose, and four‐dose vaccinated group was compared with unvaccinated patients, with lower HRs suggesting more protection. We calculated VE against COVID‐19‐associated hospitalization as (1 − HR) × 100% for each comparison. Non‐overlapping 95% CIs were considered statistically different. Analyses were further stratified by age group (18–64 and ≥65 years) and by psychiatric disorder type.

#### 2.5.4 Case–Control Test‐Negative Design

We conducted a secondary analysis among seven VISION Network partners in Colorado, Indiana, Minnesota, New York, Oregon, Texas, Utah, Washington, and Wisconsin (eTable 4 in the Supporting Information section) to estimate VE against COVID‐19‐associated hospitalization using a test‐negative design (eMethods in the Supporting Information section). This design complements the primary retrospective cohort design by minimizing biases associated with healthcare‐seeking behaviors [20, 21, 22]. The analytic sample only included hospitalized patients with a COVID‐19‐like illness discharge diagnosis, and psychiatric disorders were defined using hospital discharge diagnoses. Using multivariable logistic regression, the odds of prior receipt of two, three, and four vaccine doses (vs. unvaccinated status) were compared between SARS‐CoV‐2‐positive cases and SARS‐CoV‐2‐negative controls, stratified by psychiatric disorder status, with VE calculated as (1 − odds ratio [OR]) × 100%.

Analyses were performed using R software, Version 4.0.4, and SAS, Version 9.4. This study was reviewed and approved by institutional review boards (IRBs) at participating sites or under a reliance agreement with the IRB of Westat®. This activity was reviewed by CDC and was conducted consistent with applicable federal law and CDC policy (e.g., 45 CFR part 46.102(l) (2), 21 CFR part 56; 42 USC §241(d); 5 USC §552a; 44 USC §3501). This study presented minimal risk to participants because there was no interaction or intervention with patients; therefore, a waiver of informed consent was granted.

## 3 Results

### 3.1 Patient Characteristics

Among 2,963,172 adults in the VISION Network cohort, 2,690,200 (90.8%) did not have a known immunocompromising condition; of those, 2,436,999 (90.6%) were included (eFigure 1 in the Supporting Information section). The median age was 47 years (IQR, 33–62), 58.3% were female, 66.1% were White, 12.9% were Black, 12.0% were Hispanic, and 3.6% were Asian (Table 1). Half (50.0%) had either ≥1 underlying respiratory (10.8%) or non‐respiratory (48.1%) condition. At each patient's start date, 41.6% were unvaccinated, 35.7% had received two doses (median days since second dose, 234; IQR, 185–266), and 22.7% had received three doses (median days since third dose, 40; IQR, 19–61).

**TABLE 1:** Baseline demographic and clinical characteristics of patients in the VISION network cohort.

	Patients, no. (%)			SMD^b^
	Overall (*N* = 2,436,999)	No psychiatric disorder (*n* = 1,898,965 [77.9%])	Any psychiatric disorder^a^ (*n* = 538,034 [22.1%])	
Site				0.11
Baylor Scott & White Health	1,240,050 (50.9)	988,745 (52.1)	251,305 (46.7)	
Intermountain Healthcare	207,919 (8.5)	157,036 (8.3)	50,883 (9.5)	
Kaiser Permanente Northwest	198,032 (8.1)	146,634 (7.7)	51,398 (9.6)	
Regenstrief Institute	790,998 (32.5)	606,550 (31.9)	184,448 (34.3)	
Age, median (IQR), years	47 (33–62)	48 (33–63)	45 (32–61)	0.08
Sex				**0.29**
Male	1,015,350 (41.7)	847,781 (44.6)	167,569 (31.1)	
Female	1,421,277 (58.3)	1,050,880 (55.3)	370,397 (68.8)	
Unknown	372 (0.0)	304 (0.0)	68 (0.0)	
Race and ethnicity				**0.25**
Asian, NH	87,101 (3.6)	77,968 (4.1)	9133 (1.7)	
Black, NH	314,613 (12.9)	257,880 (13.6)	56,733 (10.5)	
Hispanic	292,191 (12.0)	239,016 (12.6)	53,175 (9.9)	
White, NH	1,610,703 (66.1)	1,213,009 (63.9)	397,694 (73.9)	
Other, NH^c^	57,888 (2.4)	47,340 (2.5)	10,548 (2.0)	
Unknown	74,503 (3.1)	63,752 (3.4)	10,751 (2.0)	
Medicaid coverage				0.11
Yes	238,245 (9.8)	173,458 (9.1)	64,787 (12.0)	
No	2,132,282 (87.5)	1,667,408 (87.8)	464,874 (86.4)	
Unknown	66,472 (2.7)	58,099 (3.1)	8373 (1.6)	
≥1 respiratory condition^d^	262,123 (10.8)	159,110 (8.4)	103,013 (19.1)	**0.32**
≥1 non‐respiratory condition (non‐psychiatric)^e^	1,172,642 (48.1)	829,153 (43.7)	343,489 (63.8)	**0.41**
No. of medical conditions (non‐psychiatric)^f^				**0.49**
0	1,218,031 (50.0)	1,038,338 (54.7)	179,693 (33.4)	
1	446,731 (18.3)	332,054 (17.5)	114,677 (21.3)	
2	287,271 (11.8)	204,134 (10.7)	83,137 (15.5)	
3	200,706 (8.2)	141,772 (7.5)	58,934 (11.0)	
4	125,966 (5.2)	86,930 (4.6)	39,036 (7.3)	
≥5	158,294 (6.5)	95,737 (5.0)	62,557 (11.6)	
mRNA COVID‐19 vaccination status^g^				**0.23**
Unvaccinated	1,013,437 (41.6)	837,185 (44.1)	176,252 (32.8)	
2 doses, 14–149 d earlier	173,316 (7.1)	126,074 (6.6)	47,242 (8.8)	
2 doses, ≥150 d earlier	697,417 (28.6)	518,382 (27.3)	179,035 (33.3)	
3 doses, 7–119 d earlier	552,829 (22.7)	417,324 (22.0)	135,505 (25.2)	

Abbreviations: IQR, interquartile range; mRNA, messenger RNA; NH, non‐Hispanic; SMD, standardized mean difference.

^a^ Any psychiatric disorder was defined as at least one mood disorder, anxiety disorder, trauma‐ or stressor‐related disorder, somatoform disorder, attention‐deficit hyperactivity disorder, eating disorder, personality disorder, dissociative or conversion disorder, or psychotic disorder.

^b^ An absolute SMD > 0.20 (shown in boldface) indicates a non‐negligible difference in a variable's distribution between patients with no psychiatric disorder and patients with any psychiatric disorder.

^c^ Other race includes American Indian or Alaska Native, Hawaiian or other Pacific Islander, other not listed, and multiple races.

^d^ Underlying respiratory conditions include asthma (overall prevalence, 5.9%), chronic obstructive pulmonary disease (3.7%), and other lung diseases (2.7%).

^e^ Underlying non‐respiratory conditions include heart failure (overall prevalence, 2.4%), ischemic heart disease (5.8%), hypertension (25.7%), other heart disease (9.8%), prior stroke (0.9%), other cerebrovascular disease (0.5%), diabetes (10.9%), other metabolic disease (30.2%), clinical obesity (12.3%), clinically underweight (0.5%), renal disease (4.7%), liver disease (2.0%), blood disorder (0.9%), dementia (0.9%), neurological/musculoskeletal disorder (7.4%), and Down syndrome (<0.1%).

^f^ The number of underlying medical conditions was defined as the sum of the number of respiratory and non‐respiratory conditions (possible range: 0–19).

^g^ For this table, vaccination status is defined at the start of each patient's eligible follow‐up period using both the number of doses received and the number of days since the most recent dose received.

**FIGURE 1:**
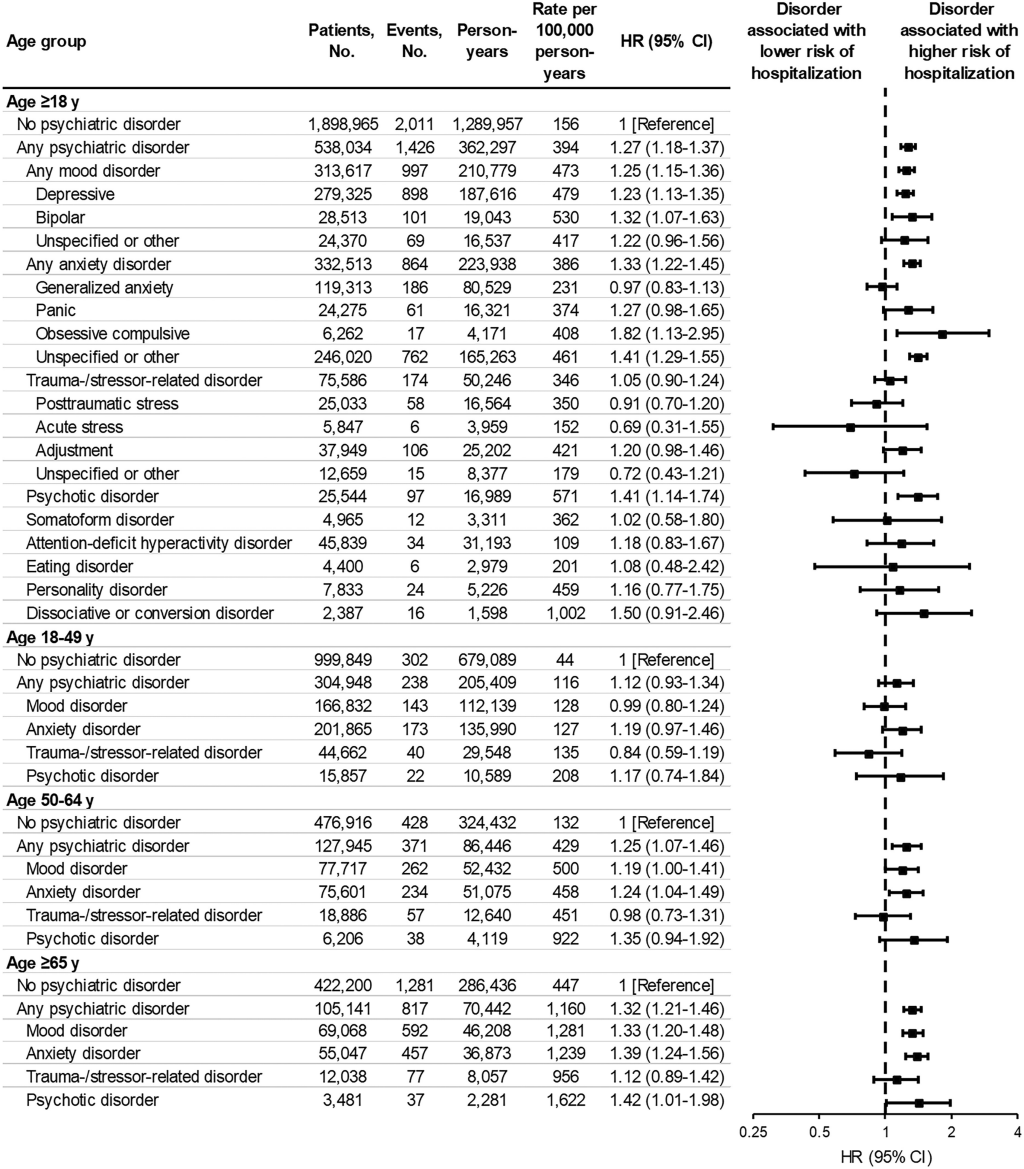
Associations between psychiatric disorders and COVID‐19‐associated hospitalization, stratified by age group. A hazard ratio (HR) > 1.0 indicates that the respective psychiatric disorder was associated with a higher risk of COVID‐19‐associated hospitalization. Each HR was obtained from a separate model comparing patients with the respective psychiatric disorder (any, mood, anxiety, trauma‐stressor‐related, or psychotic) to patients with no psychiatric disorder. HRs were adjusted for site, age (natural spline with four knots), sex (male, female, unknown), race and ethnicity (Asian, Black, Hispanic, White, other, unknown), Medicaid coverage (yes, no, unknown), underlying respiratory condition (yes, no), underlying non‐respiratory condition (yes, no), number of underlying medical conditions (square‐root transformed), number of SARS‐CoV‐2 test records documented in the patient's electronic medical record prior to the start of the study period (0, 1, 2–4, ≥5), and time‐varying mRNA COVID‐19 vaccination status (unvaccinated, two doses 14–149 days earlier, two doses ≥150 days earlier, three doses 7–119 days earlier, three doses ≥120 days earlier, four doses 7–59 days earlier, four doses ≥60 days earlier). CI, confidence interval.

Twenty‐two percent (538,034) had any psychiatric disorder, including 332,513 (13.6%) with anxiety disorders, 313,617 (12.9%) with mood disorders, 75,586 (3.1%) with trauma‐/stressor‐related disorders, 45,839 (1.9%) with attention‐deficit hyperactivity disorders, 25,544 (1.0%) with psychotic disorders, and <1% with each other disorder type (Figure 1). Among patients with ≥1 disorder, 213,018 (39.6%) had ≥2 disorder types; the most common combinations were mood and anxiety (162,679; 30.2%), anxiety and trauma‐/stressor‐related (37,304; 6.9%), and mood and trauma‐/stressor‐related (36,467; 6.8%) (data not shown). Psychiatric disorders were more common among females, non‐Hispanic White patients, patients with underlying medical condition(s), and vaccinated patients (all SMD > 0.2) (Table 1).

### 3.2 Longitudinal Follow‐Up

Among 30,564 total hospitalizations with COVID‐19‐like illness diagnoses during the study period, 19,812 (64.8%) had SARS‐CoV‐2 test results available (data not shown). This proportion was similar between patients with (64.6%) and without (65.0%) psychiatric disorders and between vaccinated (64.9%) and unvaccinated (64.7%) patients.

Among 2,436,999 patients contributing 1,652,254 person‐years, 3437 had laboratory‐confirmed COVID‐19‐associated hospitalization, corresponding to an overall incidence rate of 208 per 100,000 person‐years. The majority (3287; 95.6%) had positive molecular results, whereas 150 (4.4%) had positive antigen tests only.

### 3.3 Association Between Psychiatric Disorders and COVID‐19‐Associated Hospitalization

**FIGURE 2:**
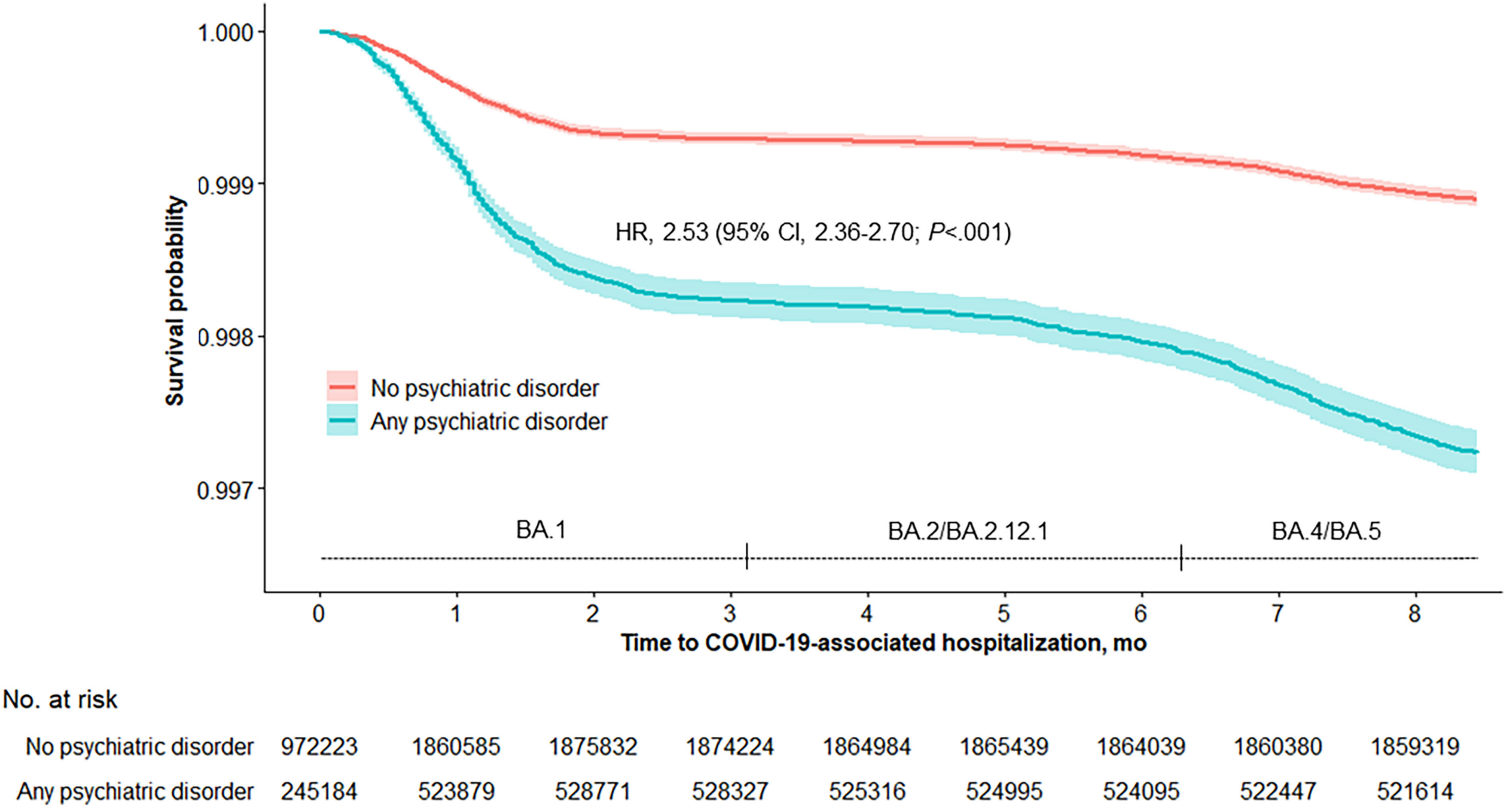
Kaplan–Meier survival curve of time to COVID‐19‐associated hospitalization, stratified by psychiatric disorder status. Time 0 is December 16, 2021, which was the earliest date a patient could start contributing eligible follow‐up. Sites had staggered entries from December 16 to 26, 2021 based on the date on which the SARS‐CoV‐2 Omicron variant first accounted for ≥50% of all sequenced specimens at each site. Individual patients could also enter the cohort at a later date if they became eligible based on a new COVID‐19 vaccination status. The large majority of patients (2,322,169; 95.3%) contributed follow‐up starting from their site‐specific start date in December 2021 through August 30, 2022. Smaller proportions entered the analytic cohort mid‐study (59,473; 2.4%) and/or were censored (53,164; 2.2%) either due to departure from the health network (33,403; 1.4%), exclusionary vaccine doses (8919; 0.4%), or death (10,842; 0.4%). Periods of estimated ≥50% BA.1 sublineage predominance (as early as December 16–26, 2021), ≥50% BA.2/BA.2.12.1 sublineage predominance (as early as March 19–24, 2022), and ≥50% BA.4/BA.5 sublineage predominance (as early as June 19–29, 2022) are displayed. The shaded areas indicate 95% confidence intervals (CIs). The unadjusted hazard ratio (HR), 95% CI, and log‐rank *p*‐value that are shown were obtained from comparing patients with any psychiatric disorder to patients with no psychiatric disorder (reference group).

The average incidence of COVID‐19‐associated hospitalization over the course of the study period was higher among patients with any (394 per 100,000 person‐years) versus no (156 per 100,000 person‐years) psychiatric disorder (unadjusted HR, 2.53; 95% CI, 2.36–2.70) (Figures 1 and 2). The absolute difference in incidence by psychiatric disorder status was highest during the earlier BA.1 Omicron sublineage predominance period. Kaplan–Meier curves for each psychiatric disorder type are provided in eFigures 2–21 in the Supporting Information section.

**FIGURE 3:**
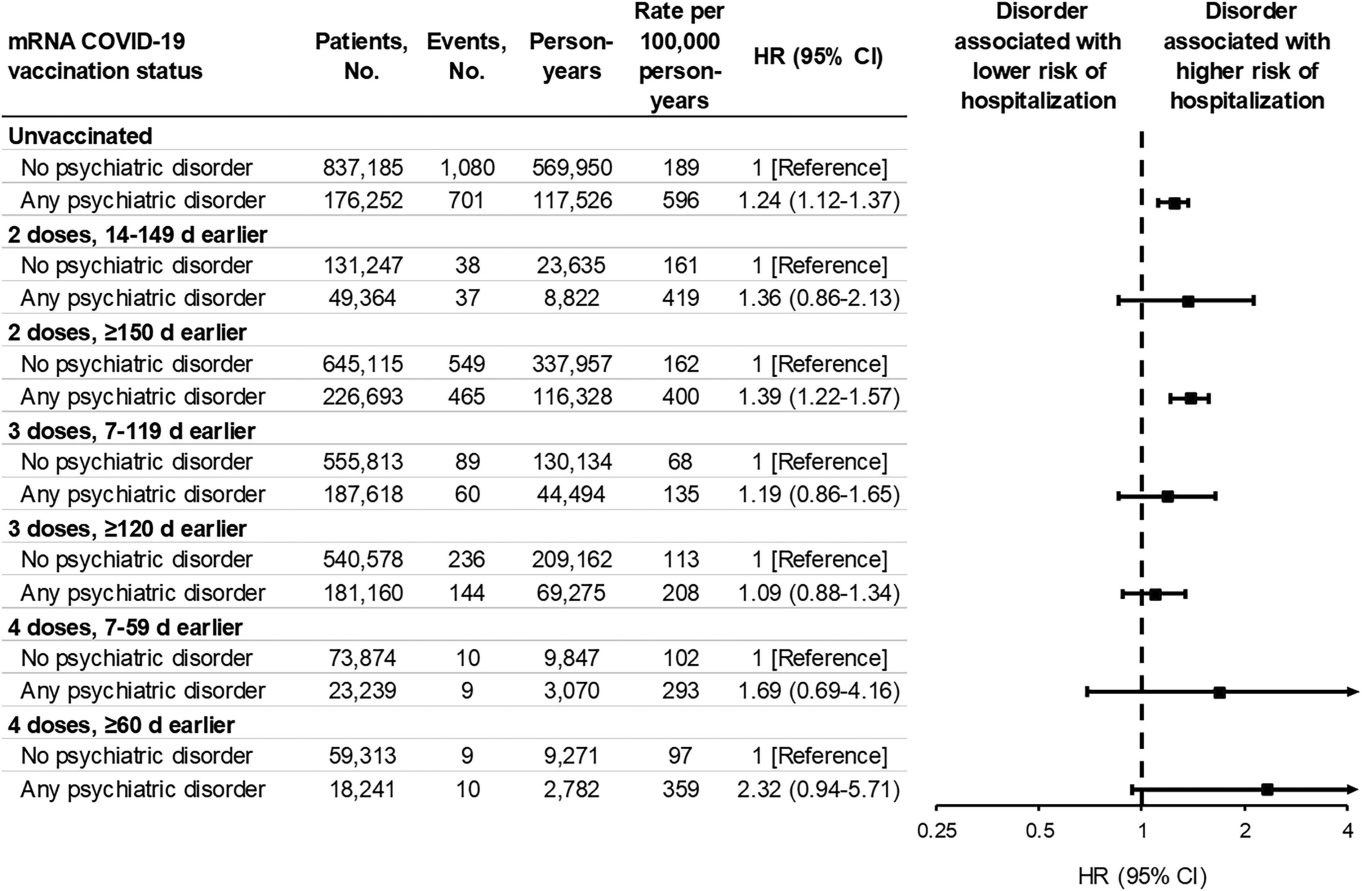
Associations between any psychiatric disorder and COVID‐19‐associated hospitalization, stratified by vaccination status. A hazard ratio (HR) > 1.0 indicates that any psychiatric disorder was associated with a higher risk of COVID‐19‐associated hospitalization. HRs were adjusted for site, age (natural spline with four knots), sex (male, female, unknown), race and ethnicity (Asian, Black, Hispanic, White, other, unknown), Medicaid coverage (yes, no, unknown), underlying respiratory condition (yes, no), underlying non‐respiratory condition (yes, no), number of underlying medical conditions (square‐root transformed), and number of SARS‐CoV‐2 test records documented in the patient's electronic medical record prior to the start of the study period (0, 1, 2–4, ≥5). All HRs except for those for four doses were obtained from the same model with the exposure variable defined by patients' vaccination status (time‐varying) and psychiatric disorder status (not time‐varying). The analysis for four doses 7–59 days earlier was limited to person‐time after April 5, 2022 among patients aged ≥50 years. The analysis for four doses ≥60 days earlier was limited to person‐time after May 28, 2022 among patients aged ≥50 years. CI, confidence interval.

In multivariable models, any (vs. no) psychiatric disorder was a significant predictor of COVID‐19‐associated hospitalization (adjusted HR, 1.27; 95% CI, 1.18–1.37) (Figure 1). Mood (adjusted HR, 1.25; 95% CI, 1.15–1.36), anxiety (adjusted HR, 1.33, 95% CI, 1.22–1.45), and psychotic (adjusted HR, 1.41; 95% CI, 1.14–1.74) disorders were each significantly associated with increased risk. Although point estimates increased with older age, an interaction between any psychiatric disorder and age group was not detected (*p* = 0.55). Associations for any disorder were similar across COVID‐19 vaccination status strata (Figure 3; test of interaction between any psychiatric disorder and vaccination status, *p* = 0.34). Associations by number and combination of disorder types did not reveal meaningful patterns (eFigure 22 in the Supporting Information section). After including each underlying condition as a separate covariate in the model, the adjusted HR for any psychiatric disorder was similar to that of various non‐psychiatric underlying conditions including asthma, obesity, and renal disease (eFigure 23 in the Supporting Information section).

### 3.4 mRNA COVID‐19 VE Against COVID‐19‐Associated Hospitalization

**FIGURE 4:**
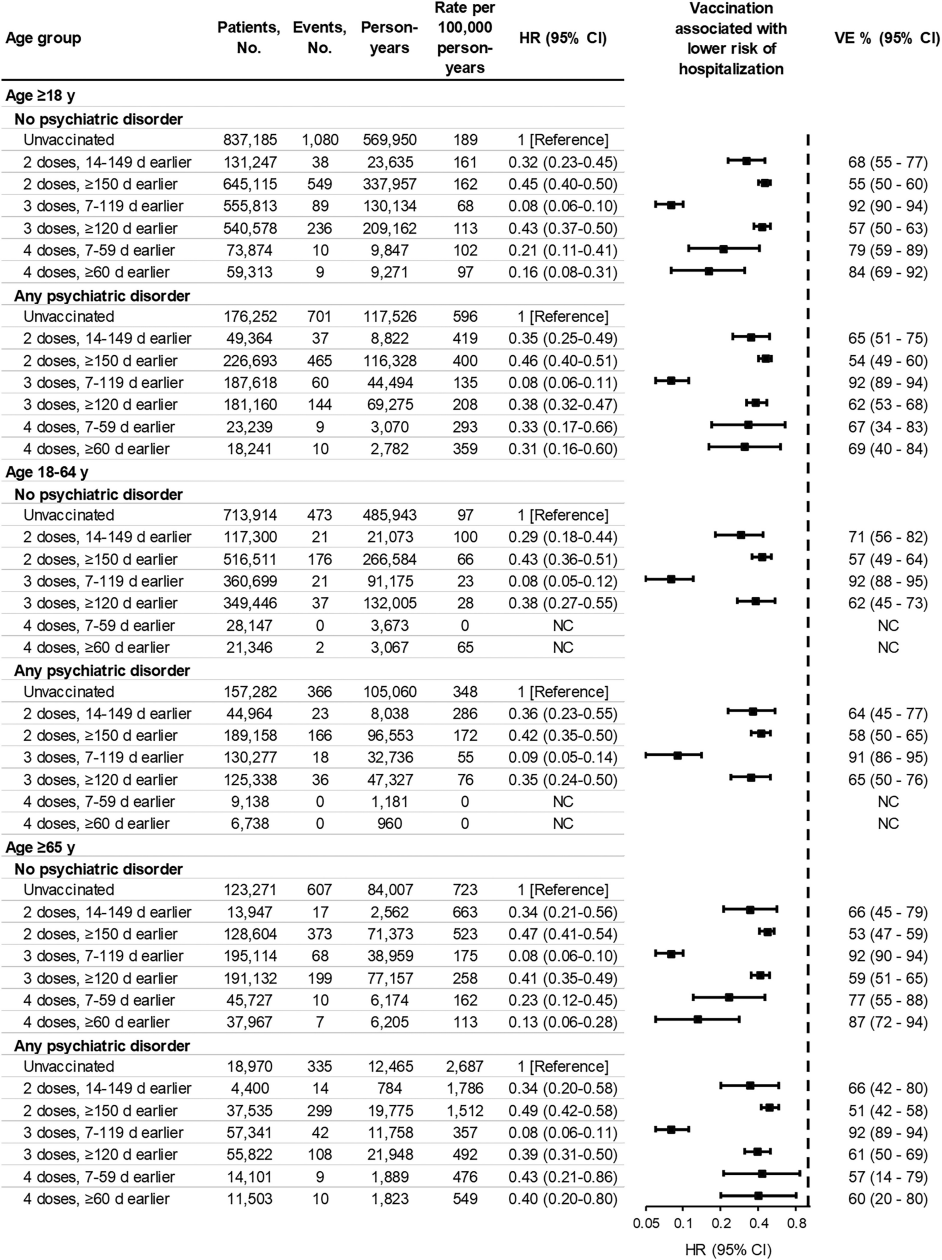
Associations between vaccination status and COVID‐19‐associated hospitalization, stratified by age group and psychiatric disorder status. A hazard ratio (HR) < 1.0 indicates that being vaccinated versus unvaccinated was associated with a lower risk of COVID‐19‐associated hospitalization. HRs were adjusted for site, age (natural spline with four knots), sex (male, female, unknown), race and ethnicity (Asian, Black, Hispanic, White, other, unknown), Medicaid coverage (yes, no, unknown), underlying respiratory condition (yes, no), underlying non‐respiratory condition (yes, no), number of underlying medical conditions (square‐root transformed), and number of SARS‐CoV‐2 test records documented in the patient's electronic medical record prior to the start of the study period (0, 1, 2–4, ≥5). For four doses 7–59 days earlier, only person‐time after April 5, 2022, among patients aged ≥50 years was analyzed. For four doses ≥60 days earlier, only person‐time after May 28, 2022, among patients aged ≥50 years was analyzed. HRs were not calculated (NC) for four doses 7–59 days earlier or four doses ≥60 days earlier in ages 18–64 due to the limited number of hospitalizations among patients aged 50–64 years in those categories. Vaccine effectiveness (VE) for prevention of COVID‐19‐associated hospitalization was estimated from HRs using the equation: VE = (1 − HR) × 100%. Vaccination status was defined as a time‐varying variable. Throughout follow‐up, 903,142 patients (37.1%) transitioned from one vaccination status to another at least once, including 289,477 (11.9%) with a new dose and 889,113 (36.5%) surpassing the cutoff of 150, 120, or 60 days since second, third, or fourth dose, respectively. At the end of follow‐up, 1,006,142 (41.3%) remained unvaccinated (patients with no psychiatric disorder, 43.8%; patients with any psychiatric disorder, 32.4%), 687,426 (28.2%) had received two doses (patients with no psychiatric disorder, 26.9%; patients with any psychiatric disorder, 32.8%) (median days since second dose, 482; IQR, 397–518), 646,315 (26.5%) had received three doses (patients with no psychiatric disorder, 25.4%; patients with any psychiatric disorder, 30.6%) (median days since third dose, 270; IQR: 243–300), and 97,113 (4.0%) had received four doses (patients with no psychiatric disorder, 3.9%; patients with any psychiatric disorder, 4.3%) (median days since fourth dose, 110; IQR: 73–134). CI, confidence interval.

The relative hazard reduction associated with being vaccinated (vs. unvaccinated) was similar between patients with any vs. no psychiatric disorder (with overlapping 95% CIs), suggesting that VE against COVID‐19‐associated hospitalization was similar (Figure 4). Among patients with no psychiatric disorder, adjusted HRs for the association between vaccination and hospitalization were 0.32 (95% CI, 0.23–0.45) at 14–149 days following second dose, 0.08 (95% CI, 0.06–0.10) at 7–119 days following third dose, and 0.21 (95% CI, 0.11–0.41) at 7–59 days following fourth dose. Corresponding VE point estimates were 68%, 92%, and 79%, respectively. Among patients with any psychiatric disorder, HRs were 0.35 (95% CI, 0.25–0.49), 0.08 (95% CI, 0.06–0.11), and 0.33 (95% CI, 0.17–0.66), corresponding to VE estimates of 65%, 92%, and 67%, respectively. This pattern was consistent among patients aged 18–64 and ≥65 years. VE for subgroups with common psychiatric disorder types are provided in eFigure 24 in the Supporting Information section.

### 3.5 Case–Control Test‐Negative Design

Characteristics of the test‐negative design sample are provided in eTable 5 in the Supporting Information section. Adjusted ORs for the association between vaccination status and symptomatic laboratory‐confirmed SARS‐CoV‐2 infection at a hospitalization (and corresponding VE estimates) were also similar between patients with any versus no psychiatric disorder (eFigure 25 in the Supporting Information section).

## 4 Discussion

In this large multi‐state EHR‐based cohort study, we found that various psychiatric disorders were independently associated with increased risk of COVID‐19‐associated hospitalization. Additionally, the protection associated with receipt of an mRNA COVID‐19 vaccine primary series or monovalent booster dose(s) was similar on a relative scale for adults with or without psychiatric disorders. This study is among the first to establish that psychiatric disorders continued to pose a risk for severe COVID‐19 during Omicron variant predominance while also providing real‐world evidence that mRNA vaccination was linked to a reduced risk of COVID‐19‐associated hospitalization in adults with psychiatric disorders. Despite higher vaccination rates and similar VE, adults with psychiatric disorders continued to face a heightened risk of hospitalization. Thus, additional strategies alongside vaccination are needed to mitigate the risk of severe COVID‐19 in this population.

The crude incidence rate of COVID‐19‐associated hospitalization was 2.5 times higher among patients with any psychiatric disorder diagnosis compared to those without. High rates were observed in patients with unspecified/other anxiety (3.0 times higher), personality (3.0 times), depressive (3.1 times), bipolar (3.4 times), psychotic (3.7 times), and dissociative/conversion (6.4 times) disorders. Associations were attenuated after adjusting for demographic and clinical characteristics including comorbidities, yet mood, anxiety, and psychotic disorders each remained associated with a 25% greater, 33% greater, and 41% greater risk of COVID‐19 hospitalization, respectively. Importantly, these effect sizes were comparable to those obtained for non‐psychiatric comorbidities including asthma, obesity, and renal disease. These findings align with a pre‐Omicron study among US veterans, which identified mood, anxiety, and psychotic disorders as risk factors for SARS‐CoV‐2 infection in adults vaccinated with an mRNA or Ad26.COV2.S primary series [11]. In contrast to that study, we found no association for posttraumatic stress disorder and a nonsignificant (yet positive) association for adjustment disorder with respect to hospitalization. Our findings are also generally consistent with those of earlier studies predating COVID‐19 vaccines [1, 2, 3, 4, 5, 23].

Based on research demonstrating that psychiatric symptoms have been associated with impaired antibody or cell‐mediated responses to numerous other vaccines, researchers have posited that psychiatric symptoms may impact COVID‐19 VE [12, 13]. Initial studies of antibody responses following COVID‐19 vaccination have had mixed results. One study found an association between depression and lower antibody positivity, while another found no association between depression or anxiety and antibody positivity [14, 24]. Antibody positivity, however, is only one measure of a multifaceted vaccine‐induced immune response, which also involves the activation of CD4+ helper T cells and CD8+ cytotoxic T cells and the development of immunological memory. In our study, VE for two‐dose and three‐dose regimens was very similar in patients with and without psychiatric disorders. For four‐dose VE (evaluated among patients aged ≥50 years starting in April 2022), point estimates were not as close but were less precise with overlapping CIs. In addition, although estimates from cohort and test‐negative design analyses differed slightly from one another, VE within each design was similar regardless of psychiatric disorder status, providing robustness to results. Overall, VE did not meaningfully differ based on psychiatric disorder status. While reassuring, it is important to note that this finding could in part stem from our use of broad diagnosis‐based definitions for psychiatric disorders. We were not able to examine more specific measures and indicators, such as symptom severity, health and sleep behaviors, inflammatory markers, and psychotropic medications, which may contribute to heterogeneity in vaccine response among patients with psychiatric disorders.

Our findings have important implications for clinical practice as well as education or communication campaigns, emphasizing the need for providers and patients to recognize psychiatric disorders as conditions that may contribute to elevated risk of severe COVID‐19 [25], in additional to traditional risk factors such as cardiac, pulmonary, or immunocompromising conditions. Healthcare professionals should strongly advise individuals with psychiatric disorders to receive COVID‐19 vaccination, ensuring that they are up to date with recommended vaccine doses. Additionally, following a positive SARS‐CoV‐2 test alongside mild‐to‐moderate symptoms, outpatient antiviral therapies such as nirmatrelvir/ritonavir can reduce the risk of progression to severe COVID‐19 disease. Close monitoring and encouragement to seek care if symptoms worsen are also critical.

This study has several limitations. First, psychiatric disorders were defined using ICD codes assigned in clinical practice and information on the severity or trajectory of specific symptoms was not available. Patients with psychiatric disorder(s) had ≥1 qualifying ICD code at ≥1 occurrence(s) during a historical period and new diagnoses during follow‐up were not examined; thus, misclassification is possible. If patients with undiagnosed psychiatric illness were classified as not having a psychiatric disorder, then associations between psychiatric disorders and hospitalization would be biased toward the null. Second, data on pharmacologic and behavioral treatments for psychiatric disorders were not available. Third, because more than one‐third of all hospitalizations with a COVID‐19‐like illness discharge diagnosis did not have SARS‐CoV‐2 test results available, incidence rates may have been underestimated. However, the proportion was similar by psychiatric disorder status and vaccination status, suggesting that possible underestimation would likely be non‐differential and not expected to bias HRs. Fourth, beyond diagnosis codes and SARS‐CoV‐2 test results, the severity of symptoms and specific reason(s) for hospitalization were not available and may have differed between patients with and without psychiatric disorders. Fifth, because patients were included regardless of prior infection, VE estimates may have been biased toward the null if prior infection was more prevalent among unvaccinated patients and was associated with some protection against reinfection or attenuation of severity if reinfected. Sixth, although we adjusted for demographic and clinical factors, residual or unmeasured confounding is possible. Apart from Medicaid status, other socioeconomic indicators were not collected. Seventh, the Ad26.COV2.S vaccine, which was targeted for populations disproportionately affected by psychiatric disorders due to the easier logistics of a single‐dose primary series in relatively transient populations (e.g., people experiencing homelessness, adult care homes), was not examined in the current analysis. Eighth, VE estimates from the retrospective cohort design could have been affected by bias related to healthcare‐seeking behaviors, which may have differed between patients with and without psychiatric disorder(s). This limitation was addressed by conducting a secondary VE analysis using the test‐negative design among hospitalized patients with COVID‐19‐like illness defined using documented discharge diagnoses. Ninth, substance use disorders, which often co‐occur with psychiatric disorders, were not examined in the current study. However, previous research has evaluated these disorders in relation to severe COVID‐19 and COVID‐19 VE [26, 27, 28, 29, 30, 31]. Tenth, data on neurodevelopmental disorders (aside from attention‐deficit hyperactivity disorder) were also not examined but have been reported in another VISION Network study [32].

## 5 Conclusions

Psychiatric disorders were associated with increased risk of COVID‐19‐associated hospitalization, yet the relative protection associated with mRNA vaccination was similar irrespective of psychiatric disorder status, underscoring the benefit of COVID‐19 vaccination in this population. Future research should continue to monitor risk of COVID‐19, severe outcomes, and real‐world COVID‐19 VE among individuals with psychiatric disorders. Since individuals with psychiatric disorders continue to disproportionately experience severe COVID‐19 health outcomes despite mRNA vaccines being similarly effective in this population, there is also a need to better characterize the drivers of increased risk and identify other effective mitigation strategies in addition to vaccination.

## Author Contributions

**Matthew Levy:** Conceptualization; Data curation; Formal analysis; Investigation; Methodology; Project administration; Supervision; Writing – original draft; Writing – review and editing. **Duck‐Hye Yang:** Conceptualization; Data curation; Formal analysis; Investigation; Methodology; Writing – original draft; Writing – review and editing. **Margaret Dunne:** Conceptualization; Data curation; Investigation; Methodology; Project administration; Writing – original draft; Writing – review and editing. **Kathleen Miley:** Conceptualization; Data curation; Investigation; Methodology; Writing – review and editing. **Stephanie Irving:** Conceptualization; Data curation; Investigation; Methodology; Writing – review and editing. **Shaun Grannis:** Conceptualization; Data curation; Investigation; Methodology; Supervision; Writing – review and editing. **Zachary Weber:** Data curation; Investigation; Writing – original draft; Writing – review and editing. **Eric P. Griggs:** Data curation; Investigation; Project administration; Writing – review and editing. **Talia Spark:** Conceptualization; Data curation; Investigation; Methodology; Writing – review and editing. **Elizabeth Bassett:** Data curation; Investigation; Writing – original draft; Writing – review and editing. **Peter Embi:** Data curation; Investigation; Supervision; Writing – review and editing. **Manjusha Gaglani:** Data curation; Investigation; Supervision; Writing – review and editing. **Karthik Natarajan:** Data curation; Investigation; Supervision; Writing – review and editing. **Nimish Valvi:** Data curation; Investigation; Writing – review and editing. **Toan Ong:** Data curation; Investigation; Supervision; Writing – review and editing. **Allison Naleway:** Data curation; Investigation; Supervision; Writing – review and editing. **Edward Stenehjem:** Data curation; Investigation; Supervision; Writing – review and editing. **Nicola Klein:** Data curation; Investigation; Supervision; Writing – review and editing. **Ruth Link‐Gelles:** Conceptualization; Data curation; Investigation; Methodology; Project administration; Supervision; Writing – review and editing. **Malini DeSilva:** Data curation; Investigation; Supervision; Writing – review and editing. **Anupam Kharbanda:** Data curation; Investigation; Supervision; Writing – review and editing. **Chandni Raiyani:** Data curation; Investigation; writing – review and editing. **Maura Beaton:** Data curation; Investigation; Writing – review and editing. **Brian Dixon:** Data curation; Investigation; Writing – review and editing. **Suchitra Rao:** Data curation; Investigation; Writing – review and editing. **Kristin Dascomb:** Data curation; Investigation; Writing – review and editing. **Palak Patel:** Data curation; Investigation; Project administration; Writing – review and editing. **Mufaddal Mamawala:** Data curation; Investigation; Writing – review and editing. **Jungmi Han:** Data curation; Investigation; Writing – review and editing. **William Fadel:** Data curation; Investigation; Writing – review and editing. **Michelle Barron:** Data curation; Investigation; Writing – review and editing. **Nancy Grisel:** Data curation; Investigation; Writing – review and editing. **Monica Dickerson:** Data curation; Investigation; Project administration; Writing – review and editing. **I‐Chia Liao:** Data curation; Investigation; Writing – review and editing. **Julie Arndorfer:** Data curation; Investigation; Writing – review and editing. **Morgan Najdowski:** Data curation; Investigation; Writing – review and editing. **Kempapura Murthy:** Data curation; Investigation; Writing – review and editing. **Caitlin Ray:** Data curation; Investigation; Project administration; Writing – review and editing. **Mark W. Tenforde:** Conceptualization; Data curation; Investigation; Methodology; Project administration; Supervision; Writing – review and editing. **Sarah Ball:** Conceptualization; Data curation; Investigation; Methodology; Project administration; Supervision; Writing – review and editing.

## Ethics Statement

This study was reviewed and approved by institutional review boards (IRBs) at participating sites or under a reliance agreement with the IRB of Westat®. This activity was reviewed by CDC and was conducted consistent with applicable federal law and CDC policy (e.g., 45 CFR part 46.102(l) (2), 21 CFR part 56; 42 USC §241(d); 5 USC §552a; 44 USC §3501).

## Consent

This study presented minimal risk to participants because there was no interaction or intervention with patients; therefore, a waiver of informed consent was granted.

## Conflicts of Interest

All authors have completed and submitted the International Committee of Medical Journal Editors form for disclosure of potential conflicts of interest. During the conduct of the study, all Westat‐ and Kaiser Permanente Northern California Division of Research‐affiliated authors reported receiving contractual support from the CDC via payments made to their respective institutions. Additionally, all authors affiliated with Baylor Scott & White Health, Children's Minnesota, Columbia University Irving Medical Center, HealthPartners Institute, Intermountain Healthcare, Kaiser Permanente Center for Health Research, Regenstrief Institute, University of Colorado Anschutz Medical Campus, and Vanderbilt University Medical Center reported receiving contractual support from the CDC during the conduct of the study, via subcontracts from Westat, Inc. with payments made to their respective institutions. Unrelated to the submitted work, the following disclosures were reported from the past 36 months: Dr. Gaglani received grants directly from CDC and from CDC via subcontracts from Abt Associates and Vanderbilt University Medical Center to her institution; Dr. Naleway received grants from Pfizer and Vir Biotechnology; Dr. Klein received grants from Pfizer, Merck, GlaxoSmithKline, and Sanofi Pasteur; Dr. Dixon reported receiving grants from CDC, NIH, AHRQ, and the U.S. Department of Veterans Affairs to his institution as well as personal fees from Elsevier and Springer Nature and consulting fees from Merck and Co.; Dr. Rao received grants from GSK; and Dr. Murthy received grants from CDC to his institution. The other authors declare no conflicts of interest.

### Peer Review

The peer review history for this article is available at https://www.webofscience.com/api/gateway/wos/peer‐review/10.1111/irv.13269.

## Supporting information


**Data S1.** Supporting Information.

## Data Availability

Data collected for this study are not available. Data sharing agreements between CDC and VISION Network partner institutions prohibit CDC from making this dataset publicly available.
