# Supplementary material for: Risk of COVID‐19 Hospitalization and Protection Associated With mRNA Vaccination Among US Adults With Psychiatric Disorders
Source: Influenza Other Respir Viruses. 2024 Mar 17;18(3):e13269. doi: 10.1111/irv.13269 (PMC10944689; doi:10.1111/irv.13269)
Supplement: Supplementary file 1 — Data S1. Supporting Information. [file IRV-18-e13269-s001.docx]

**Supplemental Material**

**eMethods**. Supplemental Methods.

**eTable 1.** Description of Partners in the VISION Network Cohort.

**eTable 2.** COVID-19–Like Illness Categories and Corresponding *International Classification of Diseases, 9th and 10th Revision* Diagnosis Codes.

**eTable 3.** Psychiatric Disorders and Corresponding *International Classification of Diseases, 9th and 10th* Revision Diagnosis Codes.

**eTable 4.** Characteristics of VISION Network Partners in the Case-Control Test-Negative Design Study.

**eTable 5.** Demographic and Clinical Characteristics of Patients in the VISION Network Test-Negative Case-Control Study.

**eFigure 1.** Flow Diagram for the Selection of Patients.

**eFigure 2.** Kaplan-Meier Survival Curve of Time to COVID-19–Associated Hospitalization Comparing Any Mood Disorder with No Psychiatric Disorder.

**eFigure 3.** Kaplan-Meier Survival Curve of Time to COVID-19–Associated Hospitalization Comparing Depressive Disorder with No Psychiatric Disorder.

**eFigure 4.** Kaplan-Meier Survival Curve of Time to COVID-19–Associated Hospitalization Comparing Bipolar Disorder with No Psychiatric Disorder.

**eFigure 5.** Kaplan-Meier Survival Curve of Time to COVID-19–Associated Hospitalization Comparing Unspecified or Other Mood Disorder with No Psychiatric Disorder.

**eFigure 6.** Kaplan-Meier Survival Curve of Time to COVID-19–Associated Hospitalization Comparing Any Anxiety Disorder with No Psychiatric Disorder.

**eFigure 7.** Kaplan-Meier Survival Curve of Time to COVID-19–Associated Hospitalization Comparing Generalized Anxiety Disorder with No Psychiatric Disorder.

**eFigure 8.** Kaplan-Meier Survival Curve of Time to COVID-19–Associated Hospitalization Comparing Panic Disorder with No Psychiatric Disorder.

**eFigure 9.** Kaplan-Meier Survival Curve of Time to COVID-19–Associated Hospitalization Comparing Obsessive Compulsive Disorder with No Psychiatric Disorder.

**eFigure 10.** Kaplan-Meier Survival Curve of Time to COVID-19–Associated Hospitalization Comparing Unspecified or Other Anxiety Disorder with No Psychiatric Disorder.

**eFigure 11.** Kaplan-Meier Survival Curve of Time to COVID-19–Associated Hospitalization Comparing Any Trauma-/Stressor-Related Disorder with No Psychiatric Disorder.

**eFigure 12.** Kaplan-Meier Survival Curve of Time to COVID-19–Associated Hospitalization Comparing Posttraumatic Stress Disorder with No Psychiatric Disorder.

**eFigure 13.** Kaplan-Meier Survival Curve of Time to COVID-19–Associated Hospitalization Comparing Acute Stress Disorder with No Psychiatric Disorder.

**eFigure 14.** Kaplan-Meier Survival Curve of Time to COVID-19–Associated Hospitalization Comparing Adjustment Disorder with No Psychiatric Disorder.

**eFigure 15.** Kaplan-Meier Survival Curve of Time to COVID-19–Associated Hospitalization Comparing Unspecified or Other Trauma-/Stressor-Related Disorder with No Psychiatric Disorder.

**eFigure 16.** Kaplan-Meier Survival Curve of Time to COVID-19–Associated Hospitalization Comparing Psychotic Disorder with No Psychiatric Disorder.

**eFigure 17.** Kaplan-Meier Survival Curve of Time to COVID-19–Associated Hospitalization Comparing Somatoform Disorder with No Psychiatric Disorder.

**eFigure 18.** Kaplan-Meier Survival Curve of Time to COVID-19–Associated Hospitalization Comparing Attention-Deficit Hyperactivity Disorder with No Psychiatric Disorder.

**eFigure 19.** Kaplan-Meier Survival Curve of Time to COVID-19–Associated Hospitalization Comparing Eating Disorder with No Psychiatric Disorder.

**eFigure 20.** Kaplan-Meier Survival Curve of Time to COVID-19–Associated Hospitalization Comparing Personality Disorder with No Psychiatric Disorder.

**eFigure 21.** Kaplan-Meier Survival Curve of Time to COVID-19–Associated Hospitalization Comparing Dissociative or Conversion Disorder with No Psychiatric Disorder.

**eFigure 22.** Associations Between Number or Combination of Psychiatric Disorder Types and COVID-19–Associated Hospitalization.

**eFigure 23.** Associations of Any Psychiatric Disorder and Other Underlying Medical Conditions with COVID-19–Associated Hospitalization.

**eFigure 24.** Associations Between Vaccination Status and COVID-19–Associated Hospitalization, Stratified by Psychiatric Disorder Type.

**eFigure 25.** Association Between COVID-19–Associated Hospitalization with Prior Vaccination, Stratified By Age Group and Psychiatric Disorder Status, Using a Case-Control Test-Negative Design.

**eMethods. Supplemental Methods.**

**Section 1: Methods of the Secondary Case-Control Test-Negative Design**

**Section 1.1: Overview**

This section highlights the methodology used to estimate the association of symptomatic laboratory-confirmed SARS-CoV-2 infection in the hospital setting with vaccination status. The secondary case-control test-negative design study was conducted during a period of ≥50% Omicron predominance among hospitalizations for patients with COVID-19–like illness. The odds of having each specific vaccination status, defined as 2 doses (14-149 days earlier and ≥150 days earlier), 3 doses (7-119 days earlier and ≥120 days earlier), and 4 doses (7-59 days earlier and ≥60 days earlier) versus unvaccinated status, was compared between SARS-CoV-2–positive cases and SARS-CoV-2–negative controls. The test-negative design can minimize biases associated with access to vaccines and healthcare seeking behaviors and has been used extensively to estimate vaccine effectiveness (VE) against medically attended influenza virus illness (1, 2). Methods for the current analysis were based on those of prior VISION Network analyses, which have been detailed elsewhere (3). Vaccination status was assigned using doses received prior to the hospitalization index date, defined as either the date of collection of a respiratory specimen associated with the most recent positive or negative SARS-CoV-2 test result before the hospital admission or the date of admission (if testing occurred only after the admission date). Molecular testing for SARS-CoV-2 performed within 14 days before through <72 hours after the hospital admission was used to define each patient’s test result. Analyses were conducted separately for each pairwise vaccination status comparison (e.g., 2 doses with 2^nd^ dose 14-149 days earlier versus unvaccinated). Both inverse propensity score weighting and covariate adjustment procedures were used to control for confounding, that is, to control for differences in characteristics between vaccinated and unvaccinated patients when estimating odds ratios (ORs) for each pairwise comparison, which were then used to estimate VE in each setting using the formula VE = [1−adjusted OR] x 100%.

**Section 1.2: Repeat Hospitalizations**

One individual could contribute more than one hospitalization during the analysis period between December 16-26, 2021 and August 30, 2022. Repeat hospital admissions for the same patient that occurred within a 30-day period (with respect to prior discharge) were combined into a single event with the earliest date used as the index date. Although data were collected and analyzed at the hospitalization level rather than at the individual patient level, repeat hospitalizations among unique patients were not expected to be highly prevalent. In general, when the percentage of repeat encounters (for patients who previously contributed an encounter) exceeds 10%, a sensitivity analysis is conducted to assess the effect of within-person correlation on ORs and confidence intervals. In this analysis of hospitalizations over approximately eight months, 5.8% of hospitalizations (4,959 of 85,065) were repeat hospitalizations among patients with a prior hospitalization (after combining repeat hospitalizations within 30 days of one another into a single event). Thus, no further action was taken.

**Section 1.3: Inverse Propensity Score Weighting**

Using established methods for estimating propensity scores within case-control studies (4), we first estimated propensity-for-vaccination scores among SARS-CoV-2 test–negative controls, with potential confounding variables used as independent variables and vaccination status as the dependent variable. Next, the fitted model was used to calculate propensity-for-vaccination scores for SARS-CoV-2–positive cases. Because each vaccinated category was compared with unvaccinated status in separate analyses, the propensity score represented the estimated probability of being in the specific vaccination category of interest versus being unvaccinated, conditional on measured covariates representing potential confounding variables. Finally, in primary multivariable regression models to estimate the association between symptomatic medically attended laboratory-confirmed SARS-CoV-2 infection and vaccination status, vaccinated patients were weighted by the inverse of their propensity to be vaccinated and unvaccinated patients were weighted by the inverse of their propensity to not be vaccinated. Inverse propensity score weighting was designed to estimate an overall average treatment effect.

Propensity to be vaccinated was estimated using boosted regression trees (BRT), a nonparametric sequential regression technique (4). Regularization settings to prevent overfitting by BRT methods were determined based on overall sample size; however, the following guidelines were followed: shallow tree depth (2-3 interaction levels), large number of trees (7,000), low learning rate (0.01), and 75% bagging.

Among a set of measured covariates that were identified as potential confounders, those included in the propensity score model were covariates empirically determined to be associated with both the outcome (case-control status) and exposure (vaccination status), with significant differences between groups defined as those with an absolute standardized mean difference >0.10. The following demographic, hospital, and medical factors were considered for inclusion: age, sex, race, ethnicity, Medicaid status, calendar date (number of days since January 1, 2021 based on hospitalization index date), geographic region (based on sub-regions defined for each site), local SARS-CoV-2 circulation on the day of each hospitalization index date, urban-rural classification of facility, hospital type, number of hospital beds, chronic respiratory condition, chronic non-respiratory condition, asthma, chronic obstructive pulmonary disease, other chronic lung disease, heart failure, ischemic heart disease, hypertension, other heart disease, stroke, other cerebrovascular disease, diabetes type 1, diabetes type 2, diabetes due to underlying conditions or other specified diabetes, other metabolic disease (excluding diabetes), clinical obesity, clinical underweight, renal disease, liver disease, blood disorder, dementia, other neurological/musculoskeletal disorder, Down syndrome, and the presence of at least one prior molecular or rapid antigen SARS-CoV-2 test record documented in the electronic medical record ≥15 days before the medical encounter index date (pre-vaccination, if vaccinated). Four covariates were included in the propensity score model regardless of their association with the outcome and exposure: age, calendar date, geographic region, and local SARS-CoV-2 circulation on the day of each medical encounter index date.

Applying best practices for inverse probability of treatment weights described by Austin and Stuart (5), the distributions of weights were examined for each vaccination status comparison. In each subgroup, outlying weights were identified at the extreme upper end of the distribution. Therefore, we truncated weights at the 99^th^ percentile for each subgroup. Propensity scores and weights were calculated using the ‘twang’ R package (6). All propensity score analyses were conducted using R version 4.1.2.

**Section 1.4: Extracting Percent Positivity Data from HHS Protect**

National laboratory testing data, including data on the state and county level, are available on the password-protected HHS Protect Public Data Hub. The laboratory testing data include viral SARS-CoV-2 laboratory test results (reverse transcription polymerase chain reaction [RT-PCR]) from over 1,000 United States laboratories and testing locations including commercial and reference laboratories, public health laboratories, hospital laboratories, and other testing locations. Data presented in HHS Protect are representative of diagnostic specimens being tested and reflect the majority of, but not all, SARS-CoV-2 laboratory-based testing conducted in the United States. Data from HHS Protect are electronic health records and do not contain personally identifiable information (see https://www.hhs.gov/sites/default/files/hhs-protect-faqs.pdf for more information).

For this analysis, daily laboratory testing data were downloaded from HHS Protect and aggregated at the county level by date of report. Using the county-level average of the daily percentage of tests that were positive during each day and the prior six days, we further aggregated and computed each date’s daily seven-day average separately for each site geographic sub-region (aggregates of counties) by taking into account county population sizes. This population-weighted daily value for each site sub-region was then assigned to each hospitalization as a measure of local SARS-CoV-2 circulation based on the hospitalization index date and site sub-region of the hospital within which the respective encounter occurred. This measure of local SARS-CoV-2 circulation was used in weighting and adjustment procedures.

**Section 1.5: Primary Outcome Model and Covariate Adjustment**

The primary outcome model to estimate the association between symptomatic medically attended laboratory-confirmed SARS-CoV-2 infection and vaccination status was a multivariable logistic regression model, with SARS-CoV-2 test result (i.e., case-control status) as the dependent variable and vaccination status as an independent variable. To calculate VE, the OR was calculated comparing the vaccinated to the unvaccinated. Hospitalizations were weighted by their inverse propensity to be vaccinated (if vaccinated) or unvaccinated (if not vaccinated). Four covariates were also directly included as additional independent variables in the regression model to account for possible residual confounding that remained after inverse propensity score weighting based on BRT modeling. The four variables were age (as a spline), calendar date (as spline), geographic region, and local SARS-CoV-2 circulation on the day of each medical encounter index date (as a spline). Spline functions for calendar date, local SARS-CoV-2 circulation, and age were defined as natural cubic splines with knots at quartiles. In addition, any other covariates with distributions that remained imbalanced between vaccinated and unvaccinated patients after inverse propensity score weighting, based on an absolute standardized mean difference >0.2, were also included directly in the respective regression model.

**Section 1.6: Subgroup Analyses**

The analyses described were conducted among hospitalizations in different subgroups. Subgroup analyses were stratified by whether or not at least one psychiatric disorder discharge diagnosis was present at the hospitalization and by age group (18-64 and ≥65 years). Propensity score weights and OR estimates for each vaccination status comparison were only calculated using hospitalizations qualifying for inclusion in the respective subgroup.

**References**

1. Foppa IM, Haber M, Ferdinands JM, et al: The case test-negative design for studies of the effectiveness of influenza vaccine. Vaccine 2013; 31:3104–3109.
2. Jackson ML, Nelson JC: The test-negative design for estimating influenza vaccine effectiveness. Vaccine 2013; 31:2165–2168.
3. Thompson MG, Stenehjem E, Grannis S, et al: Effectiveness of Covid-19 vaccines in ambulatory and inpatient care settings. N Engl J Med 2021; 385:1355–1371.
4. McCaffrey DF, Ridgeway G, Morral AR: Propensity score estimation with boosted regression for evaluating causal effects in observational studies. Psychol Methods 2004; 9:403-425.
5. Austin PC, Stuart, EA: Moving towards best practices when using inverse probability of treatment weights (IPTW) using the propensity score to estimate causal treatment effects in observational studies. Stat Med 2015; 34: 3661–3679.
6. Ridgeway G, McCaffrey D, Morral AR, et al: Toolkit for weighting and analysis of nonequivalent groups: a tutorial for the R TWANG package. Santa Monica, CA: RAND Corporation; 2022. https://www.rand.org/pubs/tools/TLA570-5.html. Accessed on 25 Jun 2023.

**eTable 1. Description of Partners in the VISION Network Cohort.**

| **Network Partner (State)** | **Baylor Scott & White Health (Texas)** | **Intermountain Healthcare (Utah)** | **Kaiser Permanente Northwest (Oregon)** | **Regenstrief Institute (Indiana)** |
| --- | --- | --- | --- | --- |
| **Inclusion criteria** | Patients with ≥1 outpatient clinic medical encounter with a primary care department healthcare provider (with ≥1 ICD discharge or encounter code) between August 26, 2020-August 25, 2021 | Patients with SelectHealth in-network insurance membership with ≥1 medical encounter (with ≥1 ICD discharge or encounter code) between August 26, 2020-August 25, 2021^a^ | Patients with in-network insurance membership as of August 26, 2021, ≥30 days of membership in the prior year, an Oregon residence, and ≥1 medical encounter between August 26, 2020-August 25, 2021 (with ≥1 ICD discharge or encounter code)^a^ | Patients living in the Indianapolis metropolitan statistical area with ≥1 medical encounter (with ≥1 ICD discharge or encounter code) between August 26, 2020-August 25, 2021^a^ |
| **Source of vaccination records** | Texas ImmTrac2, State Immunization Registry and EHRs | Utah State Immunization Information System and EHRs | Oregon Immunization Information System, Washington State Immunization Information System, claims data, and EHRs | Children and Hoosier Immunization Registry Program |
| **Lookback period for underlying medical conditions**^b^ | 1 year | 1 year | 1 year | 5 years |
| **Number of hospitals** | 26 | 22 | 37^c^ | 76 |
| **Start date** | December 16, 2021 | December 24, 2021 | December 24, 2021 | December 26, 2021 |
| **End date** | August 30, 2022 | August 30, 2022 | August 30, 2022 | August 30, 2022 |
| **≥50% Omicron BA.1 sublineage predominance** | December 16, 2021 - March 18, 2022 | December 24, 2021 - March 18, 2022 | December 24, 2021 - March 23, 2022 | December 26, 2021 - March 20, 2022 |
| **≥50% Omicron BA.2/ BA.2.12.1 sublineage predominance** | March 19 - June 21, 2022 | March 19 - June 22, 2022 | March 24 - June 28, 2022 | March 21 - June 18, 2022 |
| **≥50% Omicron BA.4/BA.5 sublineage predominance** | June 22 - August 30, 2022 | June 23 - August 30, 2022 | June 29 - August 30, 2022 | June 19 - August 30, 2022 |

Abbreviations: EHR, electronic health record; ICD, International Classification of Diseases.

^a^ Medical encounters included ambulatory visits (emergency department, urgent care, outpatient, telehealth, specialty ambulatory) and inpatient visits.

^b^ The lookback period for enumerating underlying medical conditions was relative to August 26, 2021.

^c^ The Kaiser Permanente Northwest cohort was restricted to Oregon state residents and the number of hospitals listed are for those in Oregon and Washington states. However, Oregon residents could have had hospitalizations at Kaiser Permanente hospitals outside of Oregon and Washington, which were also captured and included in analyses. Less than 3% of hospitalizations occurred at hospitals outside of Oregon and Washington.

**eTable 2. COVID-19–Like Illness Categories and Corresponding *International Classification of Diseases, 9th and 10th Revision* Diagnosis Codes.**

| **Description of Diagnosis** | **ICD-10 codes** | **ICD-9 codes** |
| --- | --- | --- |
| **COVID-19 Pneumonia** |  |  |
| Pneumonia due to SARS-associated coronavirus | J12.81 | NA |
| Pneumonia due to coronavirus disease 2019 | J12.82 | NA |
| **Influenza Pneumonia** |  |  |
| Influenza due to identified novel influenza A virus with pneumonia | J09.X1 | 488.81 |
| Influenza due to other identified influenza virus with pneumonia | J10.0* | NA |
| Influenza due to other identified influenza virus with unspecified type of pneumonia | J10.00 | 487.0 |
| Influenza due to other identified influenza virus with the same other identified influenza virus pneumonia | J10.01 | 487.0 |
| Influenza due to other identified influenza virus with other specified pneumonia | J10.08 | 487.0, 488.11 |
| Influenza due to unidentified influenza virus with pneumonia | J11.0* | NA |
| Influenza due to unidentified influenza virus with unspecified type of pneumonia | J11.00 | 487.0 |
| Influenza due to unidentified influenza virus with specified pneumonia | J11.08 | 487.0 |
| Influenza with pneumonia | NA | 487* |
| **Other Viral Pneumonia** | J12.0, J12.1, J12.3, J12.3, J12.89, J12.9 | 480* |
| **Bacterial and Other Pneumonia** |  |  |
| Streptococcus pneumoniae pneumonia | J13 | 481 |
| Hemophilus influenzae pneumonia | J14 | 482.2 |
| Other bacterial pneumonia | J15* | 482* |
| Pneumonia due to other specified organism | J16* | 483* |
| Pneumonia in infectious diseases classified elsewhere | J17 | 484* |
| Pneumonia, unspecified organism | J18* | 486 |
| **Influenza Disease** | J09.X2, J09.X3, J09.X9, J10.1, J10.2, J10.8*, J11.1, J11.2, J11.8* | 488* |
| **Acute respiratory distress syndrome** | J80 | 518.82 |
| **COPD with acute exacerbation** | J44.1 | 491.21 |
| **Asthma acute exacerbation** | J45.21, J45.22, J45.31, J45.32, J45.41, J45.42, J45.51, J45.52, J45.901, J45.902 | 493.01, 493.02, 493.11, 493.12, 493.21, 493.22, 493.91, 493.92 |
| **Respiratory failure** |  |  |
| Acute respiratory failure | J96.0* | 518.81 |
| Acute and chronic respiratory failure | J96.2* | 518.84 |
| Respiratory arrest | R09.2 | 799.1 |
| **Other acute lower respiratory tract infections** |  |  |
| Acute bronchitis | J20* | 466.0 |
| Acute bronchiolitis | J21* | 466.1* |
| Unspecified acute lower respiratory infection | J22 | 519.8 |
| Bronchitis, not specified as acute or chronic | J40 | 490 |
| COPD with acute lower respiratory infection | J44.0 | 491.22 |
| Simple and mucopurulent chronic bronchitis | J41* | 491* |
| Unspecified chronic bronchitis | J42 | 491.9 |
| Emphysema | J43* | 492* |
| Bronchiectasis | J47* | 494* |
| Abscess of lung and mediastinum | J85* | 513* |
| Gangrene and necrosis of lung | J85.0 | NA |
| Abscess of lung without pneumonia | J85.2 | 513.0 |
| Abscess of mediastinum | J85.3 | 513.1 |
| Abscess of lung with pneumonia | J85.1 | 513.0 |
| Pyothorax | J86* | 510* |
| **Acute and chronic sinusitis** | J01* , J32* | 461* , 473* |
| **Acute upper respiratory tract infections** | J00*, J02*, J03*, J04*, J05*, J06* | 460*, 462, 463, 464*, 465* |
| **Signs and symptoms of acute respiratory illness** |  |  |
| Hemoptysis | R04.2 | 786.3 |
| Cough | R05 R05.1, R05.2, R05.4, R05.8, R05.9 | 786.2 |
| Dyspnea unspecified | R06.00 | 786.09 |
| Shortness of breath | R06.02 | 786.05 |
| Acute respiratory distress | R06.03 | NA |
| Stridor | R06.1 | 786.1 |
| Wheezing | R06.2 | 786.07 |
| Other abnormalities of breathing | R06.8 | NA |
| Apnea, not elsewhere classified | R06.81 | 786.03 |
| Tachypnea, not elsewhere classified | R06.82 | 786.06 |
| Other abnormalities of breathing/ other symptoms involving head and neck | R06.89 | 784.99 |
| Other dyspnea and respiratory abnormality | NA | 786.09 |
| Other symptoms involving respiratory system and chest | NA | 786.9 |
| Chest pain on breathing/ painful respiration | R07.1 | 786.52 |
| Asphyxia and hypoxemia | R09.0* | NA |
| Asphyxia | R09.01 | 799.01 |
| Hypoxemia | R09.02 | 799.02 |
| Pleurisy | R09.1 | 511.0 |
| Respiratory arrest | R09.2 | 799.1 |
| Abnormal sputum | R09.3 | 786.4 |
| Other specified symptoms and signs involving the circulatory and respiratory systems | R09.8* | 478.19, 784.91, 786.7 |
| **Signs and symptoms of acute febrile illness** |  |  |
| Fever | R50* | NA |
| Fever presenting with conditions classified elsewhere | R50.81 | 780.61 |
| Fever unspecified | R50.9 | 780.6 |
| Chills (without fever) | R68.83 | 780.64 |
| **Signs and symptoms of acute non-respiratory illness** |  |  |
| Diarrhea | R19.7 | 787.91 |
| Disturbance of smell and taste | R43* | NA |
| Unspecified disturbances of smell and taste | R43.9 | 781.1, V41.5 |
| Headache | R51.9 | 784.0 |
| Myalgia | M79.10, M79.18 | 729.1 |
| Sepsis - symptoms and signs specifically associated with systemic inflammation and infection | R65* | 785.52 |
| Other malaise | R53.81 | 780.79 |
| Other fatigue | R53.83 | 780.79 |
| Shock, unspecified | R57.9 | 785.5 |
| Debility unspecified | NA | 799.3 |
| Altered level of consciousness / altered mental status | R41.82, R40.0, R40.1 | 780.97, 780.0* |
| Weakness | R53.1 | 780.79 |
| Nausea and Vomiting | R11.0, R11.10, R11.11, R11.15, R11.2 | 787* |
| Rash and other nonspecific skin eruption | R21* | 782.1 |
| Abdominal pain | R10.0, R10.1*, R10.2, R10.3*, R10.81*, R10.84, R10.9 | 789* |

Abbreviations: COPD, Chronic Obstructive Pulmonary Disease; ICD-10, International Classification of Diseases, 10^th^ Revision; ICD-9, International Classification of Diseases, 9^th^ Revision; NA, not applicable.

* Includes all sub-codes.

**eTable 3. Psychiatric Disorders and Corresponding *International Classification of Diseases, 9th and 10th Revision* Diagnosis Codes.**

| **Description of Diagnosis** | **ICD-10 codes** | **ICD-9 codes** |
| --- | --- | --- |
| **Mood Disorders** |  |  |
| Depressive Disorder | F32.0-F32.5, F32.8*, F32.9, F32.A, F33.0-F33.3, F33.4*, F33.8, F33.9, F34.1 | 296.2*, 296.3*, 296.82, 300.4, 311 |
| Bipolar Disorder | F30.*, F31.* | 296.0*, 296.1*, 296.4*-296.7*, 296.80, 296.81, 296.89 |
| Other or Unspecified Mood Disorder | F34.0, F34.8*, F34.9, F39 | 296.9*, 301.13 |
| **Anxiety Disorders** |  |  |
| Generalized Anxiety Disorder | F41.1 | 300.02 |
| Panic Disorder | F41.0 | 300.01 |
| Obsessive Compulsive Disorder | F42.* | 300.3 |
| Other or Unspecified Anxiety Disorder | F40.0*, F40.1*, F41.3, F41.8, F41.9 | 300.00, 300.09, 300.21-300.23 |
| **Trauma- or Stressor-Related Disorders** |  |  |
| Posttraumatic Stress Disorder | F43.1* | 309.81 |
| Acute Stress Disorder | F43.0 | 308.* |
| Adjustment Disorder | F43.2* | 309.0, 309.1, 309.24, 309.28, 309.29, 309.3, 309.4, 309.9 |
| Other or Unspecified Trauma- or Stressor-Related Disorder | F43.8, F43.9 | 309.82, 309.83, 309.89 |
| **Psychotic Disorders** |  |  |
| Schizophrenia | F20.0-F20.3, F20.5, F20.89, F20.9 | 295.0*-295.3*, 295.5*, 295.6*, 295.8*, 295.9* |
| Schizoaffective Disorder | F25.* | 295.7* |
| Delusional Disorder | F22 | 297.0-297.2, 297.8, 297.9 |
| Other or Unspecified Psychotic Disorder | F20.81, F21, F23, F24, F28, F29 | 295.4*, 297.3, 298.0, 298.1, 298.3, 298.4, 298.8, 298.9, 301.22 |
| **Somatoform Disorder** | F45.* | 300.8* |
| **Attention-Deficit Hyperactivity Disorder** | F90.* | 314.0* |
| **Eating Disorder** | F50.* | 307.1, 307.50, 307.51, 307.54, 307.59 |
| **Personality Disorder** | F60.* | 301.0, 301.20, 301.3, 301.4, 301.5*, 301.6, 301.7, 301.8*, 301.9 |
| **Dissociative or Conversion Disorder** | F44.* | 300.11-300.16, 300.19 |

Abbreviations: ICD-10, International Classification of Diseases, 10^th^ Revision; ICD-9, International Classification of Diseases, 9^th^ Revision.

* Includes all sub-codes.

**eTable 4. Characteristics of VISION Network Partners in the Case-Control Test-Negative Design Study.**

| **Network partner (state)** | **Baylor Scott & White Health (Texas)** | **Columbia University Irving Medical Center (New York)** | **HealthPartners (Minnesota and Wisconsin)** | **Intermountain Healthcare (Utah)** | **Kaiser Permanente Northwest (Oregon and Washington)** | **Regenstrief Institute (Indiana)** | **University of Colorado (Colorado)** |
| --- | --- | --- | --- | --- | --- | --- | --- |
| **No. geographic sub-regions^a^ (N=34)** | 8^b^ | 1 | 2 | 8 | 3 | 9^c^ | 3 |
| **No. hospitals (N=232)** | 26 | 3 | 10 | 22 | 57 | 102 | 12 |
| **≥50% Omicron BA.1 sublineage predominance** | December 16, 2021 - March, 18, 2022 | December 18, 2021 - March, 16, 2022 | December 25, 2021 - March, 21, 2022 | December 24, 2021 - March, 18, 2022 | December 24, 2021 - March, 23, 2022 | December 26, 2021 - March, 20, 2022 | December 19, 2021 - March, 20, 2022 |
| **≥50% Omicron BA.2/ BA.2.12.1 sublineage predominance** | March 19 - June 21, 2022 | March 17 - June 28, 2022 | March 22 - June 21, 2022 | March 19 - June 22, 2022 | March 24 - June 28, 2022 | March 21 - June 18, 2022 | March 21 - June 18, 2022 |
| **≥50% Omicron BA.4/BA.5 sublineage predominance** | June 22 - August 30, 2022 | June 29 - August 30, 2022 | June 22 - August 30, 2022 | June 23 - August 30, 2022 | June 29 - August 30, 2022 | June 19 - August 30, 2022 | June 19 - August 30, 2022 |
| **Source of vaccination records** | Texas ImmTrac2, State Immunization Registry and EHRs | New York Citywide Immunization Registry and EHRs | Minnesota Immunization Information Connection and EHRs | Utah State Immunization Information System and EHRs | Oregon Immunization Information System, Washington State Immunization Information System, claims data, and EHRs | Children and Hoosier Immunization Registry Program | Colorado Immunization Information System and EHRs |

Abbreviations: EHRs, electronic health records.

^a^ Each site defined sub-regions that represent meaningfully distinct geographic areas within their network. Sub-region values were assigned to hospitalizations based on the location of the admitting hospital and were used for purposes of adjustment for geographic region in multivariable models.

^b^ For Baylor Scott & White Health, hospitals contributing data were located in 8 of 9 total sub-regions defined.

^c^ For Regenstrief Institute, hospitals were located in 9 of 10 total sub-regions defined. A subset of hospitalizations had unknown location of hospital.

**eTable 5. Demographic and Clinical Characteristics of Patients in the VISION Network Test-Negative Case-Control Study.**

|  | **Patients, No. (%)** | | | | | |
| --- | --- | --- | --- | --- | --- | --- |
|  | **Cases (SARS-CoV-2–positive)** | | | **Controls (SARS-CoV-2–negative)** | | |
|  | **No Psychiatric Disorder** | **≥1 Psychiatric Disorder^a^** | **SMD^b^** | **No Psychiatric Disorder** | **≥1 Psychiatric Disorder^a^** | **SMD^b^** |
| All hospitalizations | 11,448 (100) | 3,808 (100) | NA | 49,661 (100) | 20,148 (100) | NA |
| Omicron sublineage predominance period |  |  | 0.08 |  |  | 0.03 |
| ≥50% BA.1 sublineage predominance | 7,539 (65.9) | 2,370 (62.2) |  | 19,862 (40.0) | 7,955 (39.5) |  |
| ≥50% BA.2/BA.2.12.1 sublineage predominance | 1,349 (11.8) | 495 (13.0) |  | 17,646 (35.5) | 7,247 (36.0) |  |
| ≥50% BA.4/BA.5 sublineage predominance | 2,560 (22.4) | 943 (24.8) |  | 12,153 (24.5) | 4,946 (24.5) |  |
| Sequence of hospitalizations per person |  |  | 0.07 |  |  | 0.07 |
| First hospitalization during Omicron predominance | 11,115 (97.1) | 3,646 (95.7) |  | 46,723 (94.1) | 18,622 (92.4) |  |
| Repeat hospitalization >30 days after prior discharge | 333 (2.9) | 162 (4.3) |  | 2,938 (5.9) | 1,526 (7.6) |  |
| Site |  |  | 0.31 |  |  | 0.39 |
| Baylor Scott & White Health | 3,244 (28.3) | 1,304 (34.2) |  | 12,587 (25.3) | 6,093 (30.2) |  |
| Columbia University | 605 (5.3) | 256 (6.7) |  | 2,808 (5.7) | 1,321 (6.6) |  |
| HealthPartners | 501 (4.4) | 252 (6.6) |  | 2,934 (5.9) | 1,988 (9.9) |  |
| Intermountain Healthcare | 1,373 (12.0) | 410 (10.8) |  | 4,836 (9.7) | 2,172 (10.8) |  |
| Kaiser Permanente Northwest | 540 (4.7) | 212 (5.6) |  | 3,042 (6.1) | 1,588 (7.9) |  |
| Regenstrief Institute | 4,389 (38.3) | 947 (24.9) |  | 20,021 (40.3) | 4,609 (22.9) |  |
| University of Colorado | 796 (7.0) | 427 (11.2) |  | 3,433 (6.9) | 2,377 (11.8) |  |
| Age group, y |  |  | 0.14 |  |  | 0.22 |
| 18-49 | 1,922 (16.8) | 729 (19.1) |  | 9,326 (18.8) | 4,778 (23.7) |  |
| 50-64 | 2,517 (22.0) | 931 (24.4) |  | 10,548 (21.2) | 5,100 (25.3) |  |
| 65-74 | 2,500 (21.8) | 908 (23.8) |  | 11,224 (22.6) | 4,675 (23.2) |  |
| 75-84 | 2,657 (23.2) | 787 (20.7) |  | 11,171 (22.5) | 3,636 (18.0) |  |
| ≥85 | 1,852 (16.2) | 453 (11.9) |  | 7,392 (14.9) | 1,959 (9.7) |  |
| Sex |  |  | 0.24 |  |  | 0.27 |
| Male | 6,145 (53.7) | 1,582 (41.5) |  | 24,937 (50.2) | 7,467 (37.1) |  |
| Female | 5,303 (46.3) | 2,226 (58.5) |  | 24,724 (49.8) | 12,681 (62.9) |  |
| Race and ethnicity |  |  | 0.22 |  |  | 0.29 |
| White, NH | 7,279 (63.6) | 2,786 (73.2) |  | 31,736 (63.9) | 14,979 (74.3) |  |
| Black, NH | 1,523 (13.3) | 393 (10.3) |  | 6,614 (13.3) | 2,160 (10.7) |  |
| Hispanic | 1,223 (10.7) | 398 (10.5) |  | 4,838 (9.7) | 1,759 (8.7) |  |
| Other^c^, NH | 668 (5.8) | 142 (3.7) |  | 2,887 (5.8) | 739 (3.7) |  |
| Unknown | 755 (6.6) | 89 (2.3) |  | 3,586 (7.2) | 511 (2.5) |  |
| Medicaid status |  |  | 0.17 |  |  | 0.21 |
| Yes | 1,734 (15.1) | 802 (21.1) |  | 7,990 (16.1) | 4,812 (23.9) |  |
| No | 9,405 (82.2) | 2,920 (76.7) |  | 40,264 (81.1) | 14,822 (73.6) |  |
| Unknown | 309 (2.7) | 86 (2.3) |  | 1,407 (2.8) | 514 (2.6) |  |
| mRNA COVID-19 vaccination status at hospitalization |  |  | 0.23 |  |  | 0.12 |
| Unvaccinated | 6,763 (59.1) | 2,039 (53.5) |  | 18,617 (37.5) | 7,041 (34.9) |  |
| 2 doses, 14-149 days earlier | 244 (2.1) | 72 (1.9) |  | 1,103 (2.2) | 549 (2.7) |  |
| 2 doses, ≥150 days earlier | 2,587 (22.6) | 1,024 (26.9) |  | 12,277 (24.7) | 5,487 (27.2) |  |
| 3 doses, 7-119 days earlier | 542 (4.7) | 206 (5.4) |  | 6,617 (13.3) | 2,719 (13.5) |  |
| 3 doses, ≥120 days earlier | 1,147 (10.0) | 411 (10.8) |  | 9,546 (19.2) | 3,773 (18.7) |  |
| 4 doses, 7-59 days earlier | 66 (0.6) | 18 (0.5) |  | 792 (1.6) | 304 (1.5) |  |
| 4 doses, ≥60 days earlier | 99 (0.9) | 38 (1.0) |  | 709 (1.4) | 275 (1.4) |  |
| ≥1 chronic respiratory condition^d^ |  |  | 0.26 |  |  | 0.29 |
| Yes | 6,868 (60.0) | 2,752 (72.3) |  | 25,176 (50.7) | 13,025 (64.6) |  |
| No | 4,580 (40.0) | 1,056 (27.7) |  | 24,485 (49.3) | 7,123 (35.4) |  |
| ≥1 chronic non-respiratory condition^e^ |  |  | 0.47 |  |  | 0.45 |
| Yes | 9,254 (80.8) | 3,640 (95.6) |  | 39,814 (80.2) | 19,083 (94.7) |  |
| No | 2,194 (19.2) | 168 (4.4) |  | 9,847 (19.8) | 1,065 (5.3) |  |

Abbreviations: IQR, interquartile range; NA, not applicable; NH, non-Hispanic; SMD, standardized mean difference.

^a^ Any psychiatric disorder was defined as at least one hospital discharge diagnosis for a mood disorder, anxiety disorder, trauma- or stressor-related disorder, somatoform disorder, attention-deficit hyperactivity disorder, eating disorder, personality disorder, dissociative or conversion disorder, or psychotic disorder.

^b^ An absolute SMD >0.20 indicates a non-negligible difference in a variable’s distribution between patients with no psychiatric disorder and patients with any psychiatric disorder.

^c^ Other race includes American Indian or Alaska Native, Asian, Hawaiian or other Pacific Islander, other not listed, and multiple races.

^d^ Underlying respiratory conditions were defined using hospital discharge diagnoses and included asthma, chronic obstructive pulmonary disease, and other lung disease.

^e^ Underlying non-respiratory conditions were defined using hospital discharge diagnoses and included heart failure, ischemic heart disease, hypertension, other heart disease, prior stroke, other cerebrovascular disease, diabetes, other metabolic disease, clinical obesity, clinically underweight, renal disease, liver disease, blood disorder, dementia, neurological/musculoskeletal disorder, and Down's syndrome.

**eFigure 1. Flow Diagram for the Selection of Patients.**


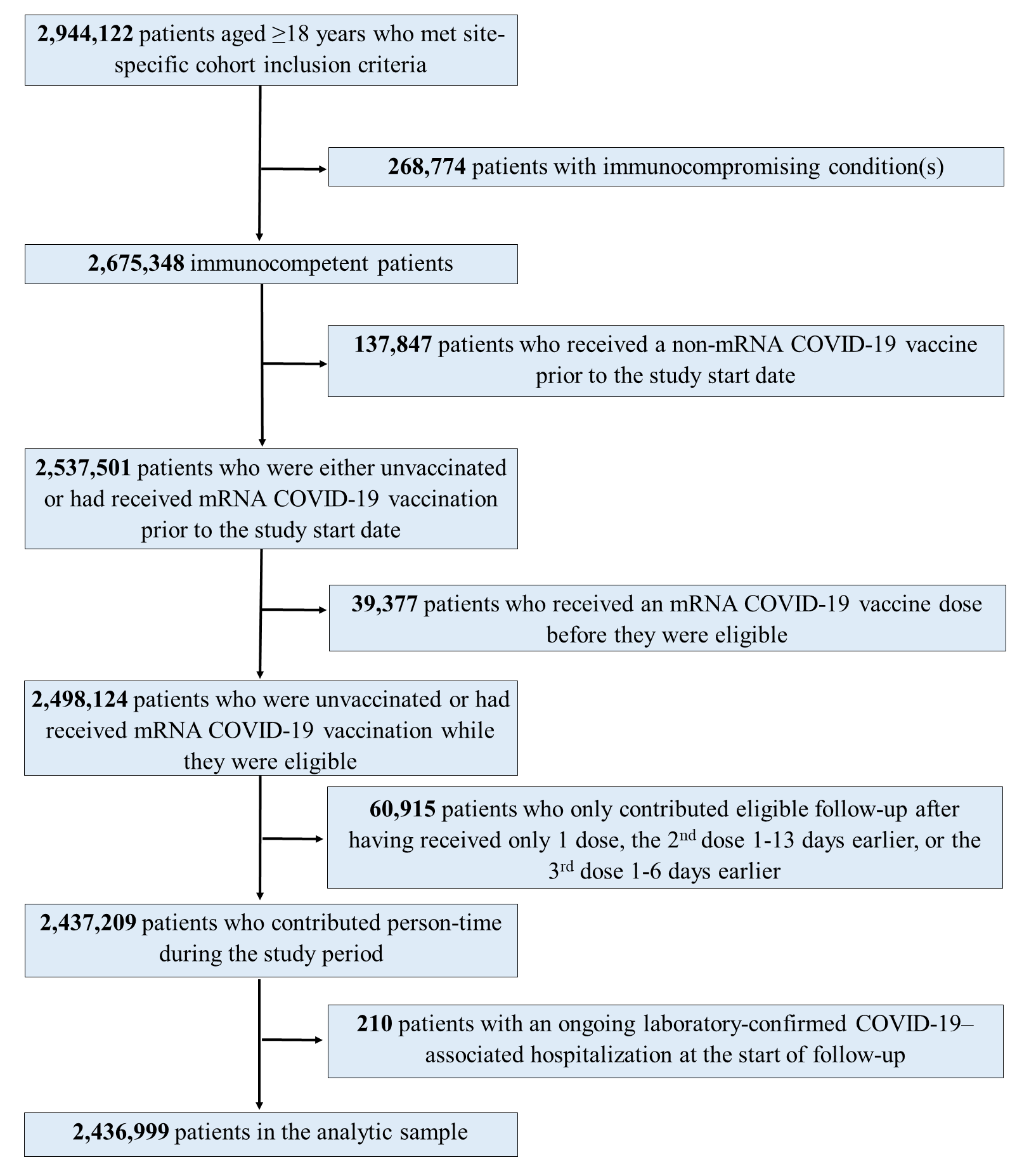


**eFigure 2. Kaplan-Meier Survival Curve of Time to COVID-19–Associated Hospitalization Comparing Any Mood Disorder with No Psychiatric Disorder.**


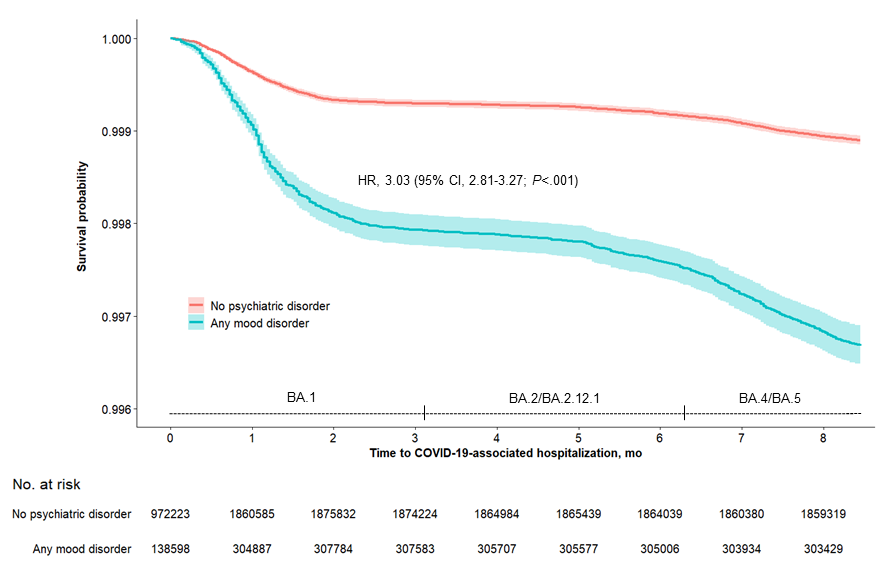


Time 0 is December 16, 2021, which was the earliest date a patient could start contributing eligible follow-up. Sites had staggered entries from December 16-26, 2021 based on the date on which the SARS-CoV-2 Omicron variant first accounted for ≥50% of all sequenced specimens at each site. Individual patients could also enter the cohort at a later date if they became eligible based on a new COVID-19 vaccination status. Periods of estimated ≥50% BA.1 sublineage predominance (as early as December 16-26, 2021), ≥50% BA.2/BA.2.12.1 sublineage predominance (as early as March 19-24, 2022), and ≥50% BA.4/BA.5 sublineage predominance (as early as June 19-29, 2022) are displayed. The shaded areas indicate 95% confidence intervals (CIs). The unadjusted hazard ratio (HR), 95% CI, and log-rank p-value that are shown were obtained from comparing patients with any mood disorder to patients with no psychiatric disorder (reference group).

**eFigure 3. Kaplan-Meier Survival Curve of Time to COVID-19–Associated Hospitalization Comparing Depressive Disorder with No Psychiatric Disorder.**


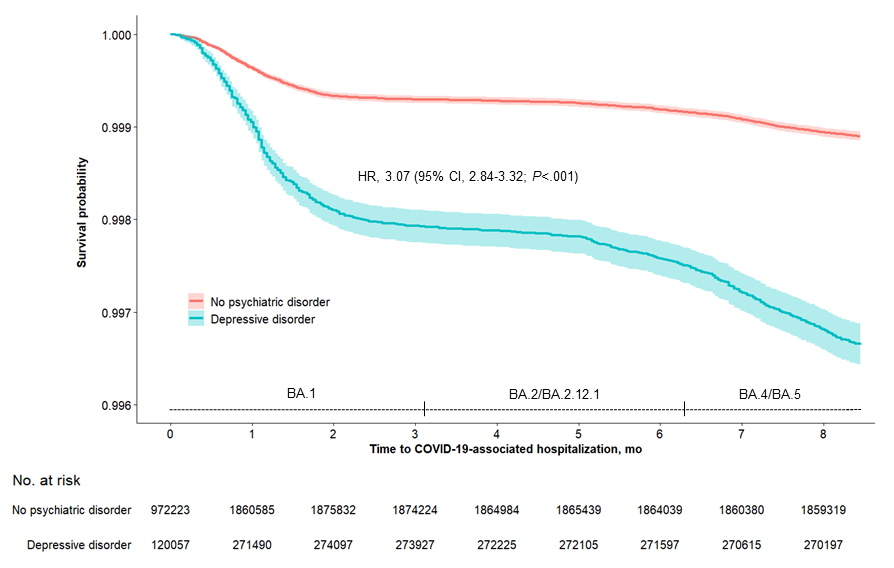


Time 0 is December 16, 2021, which was the earliest date a patient could start contributing eligible follow-up. Sites had staggered entries from December 16-26, 2021 based on the date on which the SARS-CoV-2 Omicron variant first accounted for ≥50% of all sequenced specimens at each site. Individual patients could also enter the cohort at a later date if they became eligible based on a new COVID-19 vaccination status. Periods of estimated ≥50% BA.1 sublineage predominance (as early as December 16-26, 2021), ≥50% BA.2/BA.2.12.1 sublineage predominance (as early as March 19-24, 2022), and ≥50% BA.4/BA.5 sublineage predominance (as early as June 19-29, 2022) are displayed. The shaded areas indicate 95% confidence intervals (CIs). The unadjusted hazard ratio (HR), 95% CI, and log-rank p-value that are shown were obtained from comparing patients with a depressive disorder to patients with no psychiatric disorder (reference group).

**eFigure 4. Kaplan-Meier Survival Curve of Time to COVID-19–Associated Hospitalization Comparing Bipolar Disorder with No Psychiatric Disorder.**


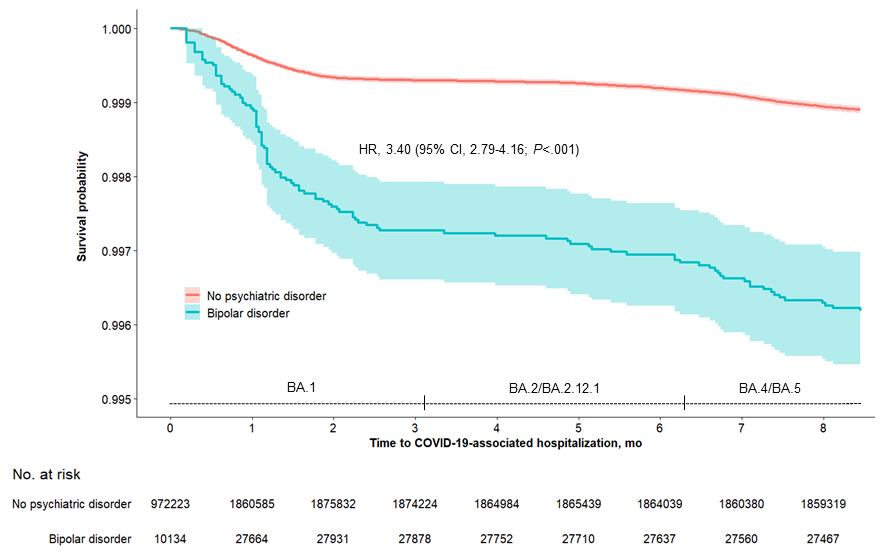


Time 0 is December 16, 2021, which was the earliest date a patient could start contributing eligible follow-up. Sites had staggered entries from December 16-26, 2021 based on the date on which the SARS-CoV-2 Omicron variant first accounted for ≥50% of all sequenced specimens at each site. Individual patients could also enter the cohort at a later date if they became eligible based on a new COVID-19 vaccination status. Periods of estimated ≥50% BA.1 sublineage predominance (as early as December 16-26, 2021), ≥50% BA.2/BA.2.12.1 sublineage predominance (as early as March 19-24, 2022), and ≥50% BA.4/BA.5 sublineage predominance (as early as June 19-29, 2022) are displayed. The shaded areas indicate 95% confidence intervals (CIs). The unadjusted hazard ratio (HR), 95% CI, and log-rank p-value that are shown were obtained from comparing patients with bipolar disorder to patients with no psychiatric disorder (reference group).

**eFigure 5. Kaplan-Meier Survival Curve of Time to COVID-19–Associated Hospitalization Comparing Unspecified or Other Mood Disorder with No Psychiatric Disorder.**


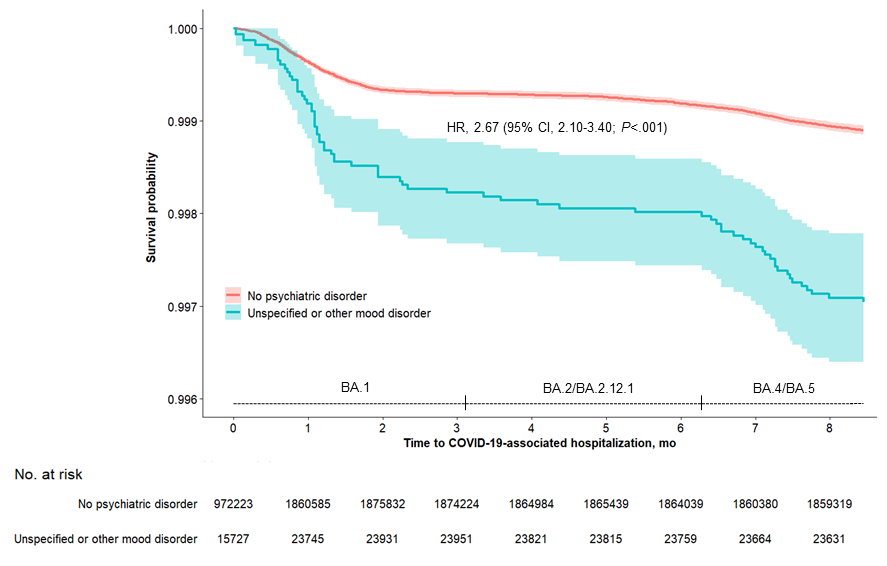


Time 0 is December 16, 2021, which was the earliest date a patient could start contributing eligible follow-up. Sites had staggered entries from December 16-26, 2021 based on the date on which the SARS-CoV-2 Omicron variant first accounted for ≥50% of all sequenced specimens at each site. Individual patients could also enter the cohort at a later date if they became eligible based on a new COVID-19 vaccination status. Periods of estimated ≥50% BA.1 sublineage predominance (as early as December 16-26, 2021), ≥50% BA.2/BA.2.12.1 sublineage predominance (as early as March 19-24, 2022), and ≥50% BA.4/BA.5 sublineage predominance (as early as June 19-29, 2022) are displayed. The shaded areas indicate 95% confidence intervals (CIs). The unadjusted hazard ratio (HR), 95% CI, and log-rank p-value that are shown were obtained from comparing patients with an unspecified or other mood disorder to patients with no psychiatric disorder (reference group).

**eFigure 6. Kaplan-Meier Survival Curve of Time to COVID-19–Associated Hospitalization Comparing Any Anxiety Disorder with No Psychiatric Disorder.**


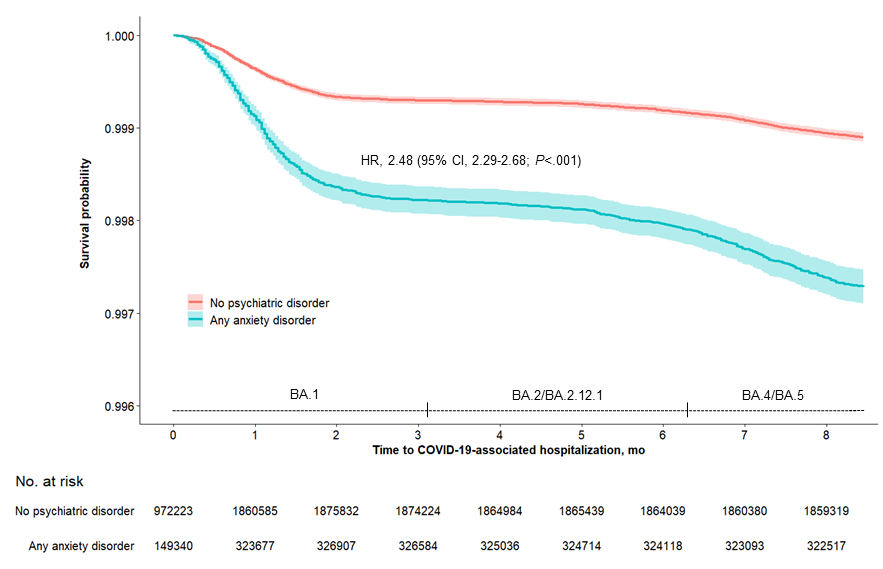


Time 0 is December 16, 2021, which was the earliest date a patient could start contributing eligible follow-up. Sites had staggered entries from December 16-26, 2021 based on the date on which the SARS-CoV-2 Omicron variant first accounted for ≥50% of all sequenced specimens at each site. Individual patients could also enter the cohort at a later date if they became eligible based on a new COVID-19 vaccination status. Periods of estimated ≥50% BA.1 sublineage predominance (as early as December 16-26, 2021), ≥50% BA.2/BA.2.12.1 sublineage predominance (as early as March 19-24, 2022), and ≥50% BA.4/BA.5 sublineage predominance (as early as June 19-29, 2022) are displayed. The shaded areas indicate 95% confidence intervals (CIs). The unadjusted hazard ratio (HR), 95% CI, and log-rank p-value that are shown were obtained from comparing patients with any anxiety disorder to patients with no psychiatric disorder (reference group).

**eFigure 7. Kaplan-Meier Survival Curve of Time to COVID-19–Associated Hospitalization Comparing Generalized Anxiety Disorder with No Psychiatric Disorder.**


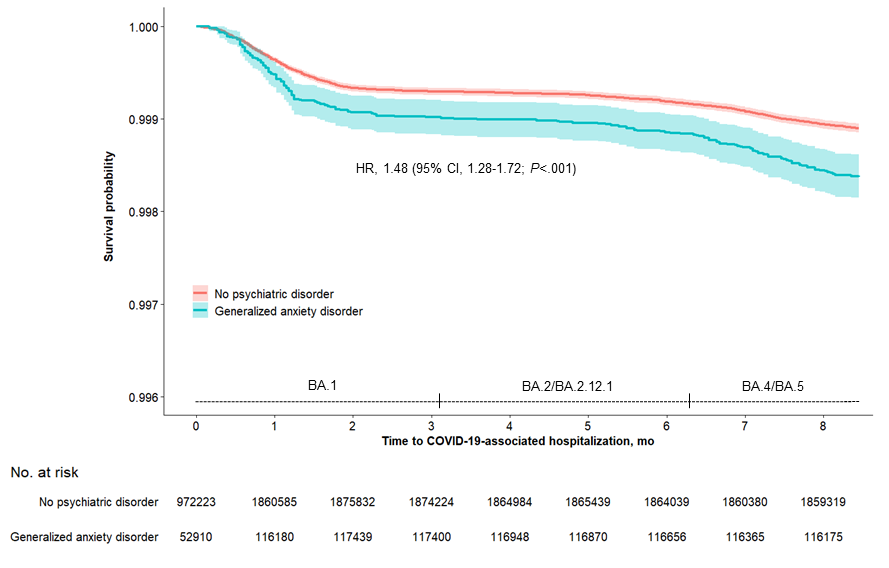


Time 0 is December 16, 2021, which was the earliest date a patient could start contributing eligible follow-up. Sites had staggered entries from December 16-26, 2021 based on the date on which the SARS-CoV-2 Omicron variant first accounted for ≥50% of all sequenced specimens at each site. Individual patients could also enter the cohort at a later date if they became eligible based on a new COVID-19 vaccination status. Periods of estimated ≥50% BA.1 sublineage predominance (as early as December 16-26, 2021), ≥50% BA.2/BA.2.12.1 sublineage predominance (as early as March 19-24, 2022), and ≥50% BA.4/BA.5 sublineage predominance (as early as June 19-29, 2022) are displayed. The shaded areas indicate 95% confidence intervals (CIs). The unadjusted hazard ratio (HR), 95% CI, and log-rank p-value that are shown were obtained from comparing patients with generalized anxiety disorder to patients with no psychiatric disorder (reference group).

**eFigure 8. Kaplan-Meier Survival Curve of Time to COVID-19–Associated Hospitalization Comparing Panic Disorder with No Psychiatric Disorder.**


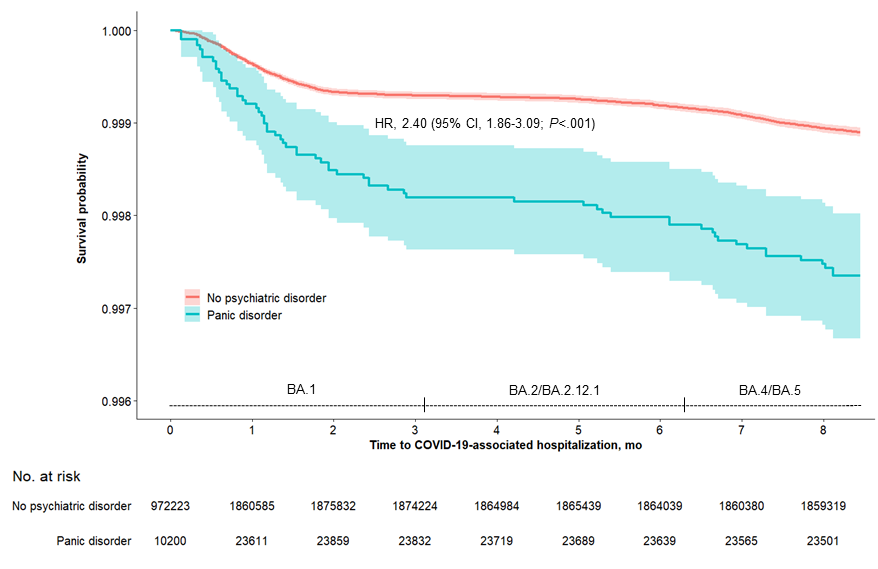


Time 0 is December 16, 2021, which was the earliest date a patient could start contributing eligible follow-up. Sites had staggered entries from December 16-26, 2021 based on the date on which the SARS-CoV-2 Omicron variant first accounted for ≥50% of all sequenced specimens at each site. Individual patients could also enter the cohort at a later date if they became eligible based on a new COVID-19 vaccination status. Periods of estimated ≥50% BA.1 sublineage predominance (as early as December 16-26, 2021), ≥50% BA.2/BA.2.12.1 sublineage predominance (as early as March 19-24, 2022), and ≥50% BA.4/BA.5 sublineage predominance (as early as June 19-29, 2022) are displayed. The shaded areas indicate 95% confidence intervals (CIs). The unadjusted hazard ratio (HR), 95% CI, and log-rank p-value that are shown were obtained from comparing patients with panic disorder to patients with no psychiatric disorder (reference group).

**eFigure 9. Kaplan-Meier Survival Curve of Time to COVID-19–Associated Hospitalization Comparing Obsessive Compulsive Disorder with No Psychiatric Disorder.**


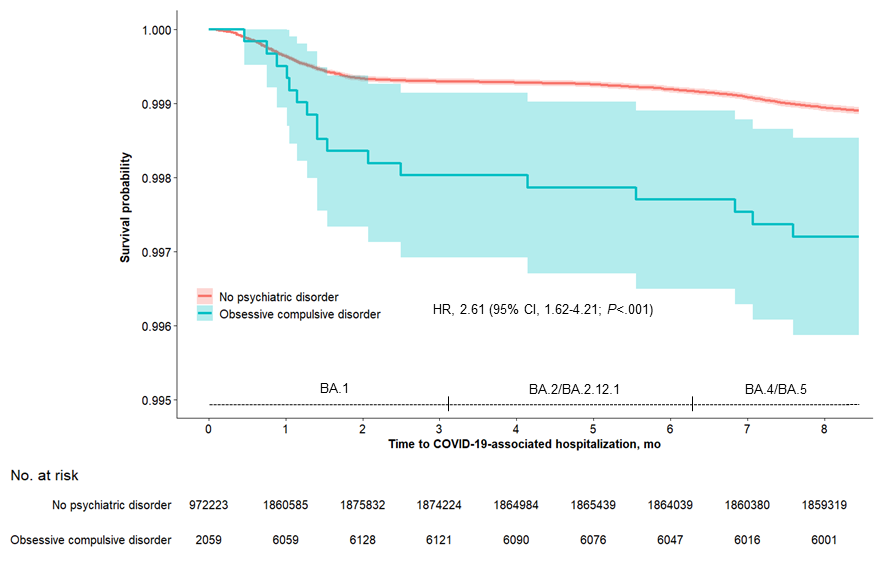


Time 0 is December 16, 2021, which was the earliest date a patient could start contributing eligible follow-up. Sites had staggered entries from December 16-26, 2021 based on the date on which the SARS-CoV-2 Omicron variant first accounted for ≥50% of all sequenced specimens at each site. Individual patients could also enter the cohort at a later date if they became eligible based on a new COVID-19 vaccination status. Periods of estimated ≥50% BA.1 sublineage predominance (as early as December 16-26, 2021), ≥50% BA.2/BA.2.12.1 sublineage predominance (as early as March 19-24, 2022), and ≥50% BA.4/BA.5 sublineage predominance (as early as June 19-29, 2022) are displayed. The shaded areas indicate 95% confidence intervals (CIs). The unadjusted hazard ratio (HR), 95% CI, and log-rank p-value that are shown were obtained from comparing patients with obsessive compulsive disorder to patients with no psychiatric disorder (reference group).

**eFigure 10. Kaplan-Meier Survival Curve of Time to COVID-19–Associated Hospitalization Comparing Unspecified or Other Anxiety Disorder with No Psychiatric Disorder.**


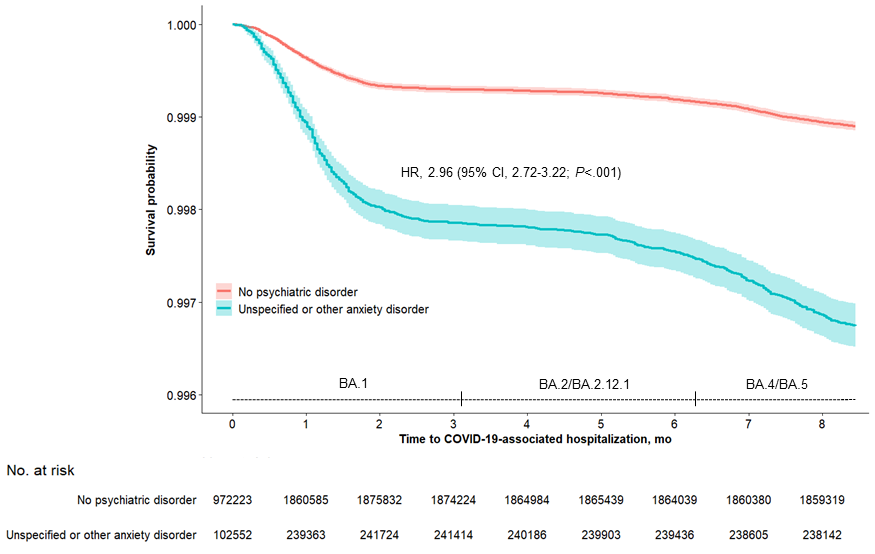


Time 0 is December 16, 2021, which was the earliest date a patient could start contributing eligible follow-up. Sites had staggered entries from December 16-26, 2021 based on the date on which the SARS-CoV-2 Omicron variant first accounted for ≥50% of all sequenced specimens at each site. Individual patients could also enter the cohort at a later date if they became eligible based on a new COVID-19 vaccination status. Periods of estimated ≥50% BA.1 sublineage predominance (as early as December 16-26, 2021), ≥50% BA.2/BA.2.12.1 sublineage predominance (as early as March 19-24, 2022), and ≥50% BA.4/BA.5 sublineage predominance (as early as June 19-29, 2022) are displayed. The shaded areas indicate 95% confidence intervals (CIs). The unadjusted hazard ratio (HR), 95% CI, and log-rank p-value that are shown were obtained from comparing patients with an unspecified or other anxiety disorder to patients with no psychiatric disorder (reference group).

**eFigure 11. Kaplan-Meier Survival Curve of Time to COVID-19–Associated Hospitalization Comparing Any Trauma-/Stressor-Related Disorder with No Psychiatric Disorder.**


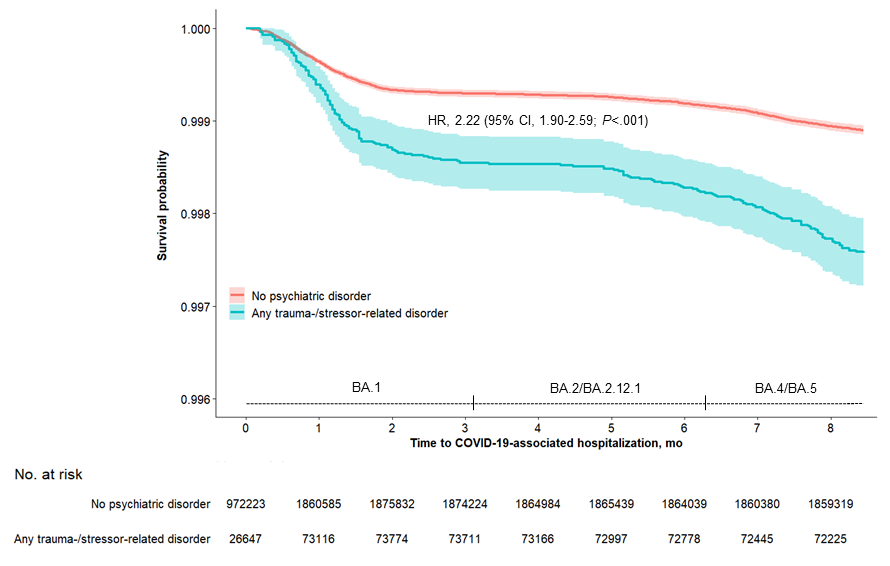


Time 0 is December 16, 2021, which was the earliest date a patient could start contributing eligible follow-up. Sites had staggered entries from December 16-26, 2021 based on the date on which the SARS-CoV-2 Omicron variant first accounted for ≥50% of all sequenced specimens at each site. Individual patients could also enter the cohort at a later date if they became eligible based on a new COVID-19 vaccination status. Periods of estimated ≥50% BA.1 sublineage predominance (as early as December 16-26, 2021), ≥50% BA.2/BA.2.12.1 sublineage predominance (as early as March 19-24, 2022), and ≥50% BA.4/BA.5 sublineage predominance (as early as June 19-29, 2022) are displayed. The shaded areas indicate 95% confidence intervals (CIs). The unadjusted hazard ratio (HR), 95% CI, and log-rank p-value that are shown were obtained from comparing patients with any trauma-/stressor-related disorder to patients with no psychiatric disorder (reference group).

**eFigure 12. Kaplan-Meier Survival Curve of Time to COVID-19–Associated Hospitalization Comparing Posttraumatic Stress Disorder with No Psychiatric Disorder.**


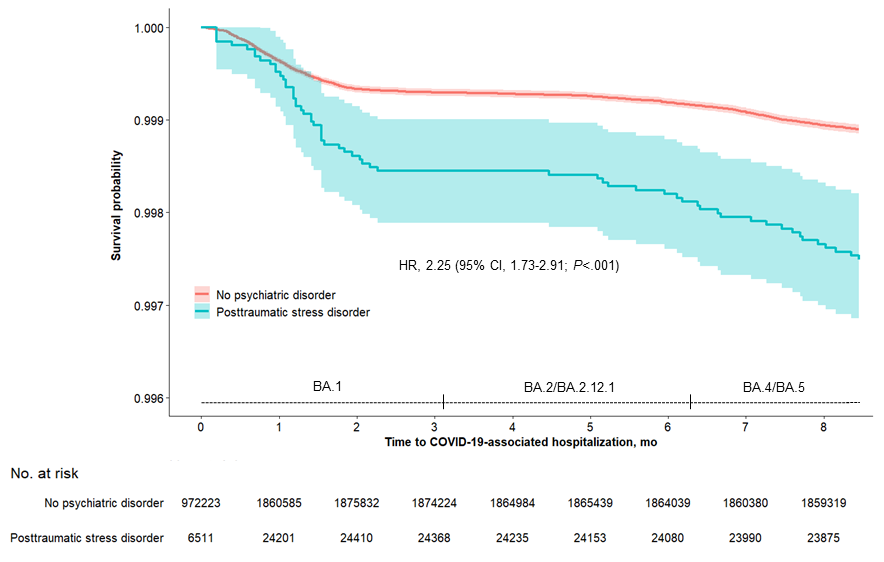


Time 0 is December 16, 2021, which was the earliest date a patient could start contributing eligible follow-up. Sites had staggered entries from December 16-26, 2021 based on the date on which the SARS-CoV-2 Omicron variant first accounted for ≥50% of all sequenced specimens at each site. Individual patients could also enter the cohort at a later date if they became eligible based on a new COVID-19 vaccination status. Periods of estimated ≥50% BA.1 sublineage predominance (as early as December 16-26, 2021), ≥50% BA.2/BA.2.12.1 sublineage predominance (as early as March 19-24, 2022), and ≥50% BA.4/BA.5 sublineage predominance (as early as June 19-29, 2022) are displayed. The shaded areas indicate 95% confidence intervals (CIs). The unadjusted hazard ratio (HR), 95% CI, and log-rank p-value that are shown were obtained from comparing patients with posttraumatic stress disorder to patients with no psychiatric disorder (reference group).

**eFigure 13. Kaplan-Meier Survival Curve of Time to COVID-19–Associated Hospitalization Comparing Acute Stress Disorder with No Psychiatric Disorder.**


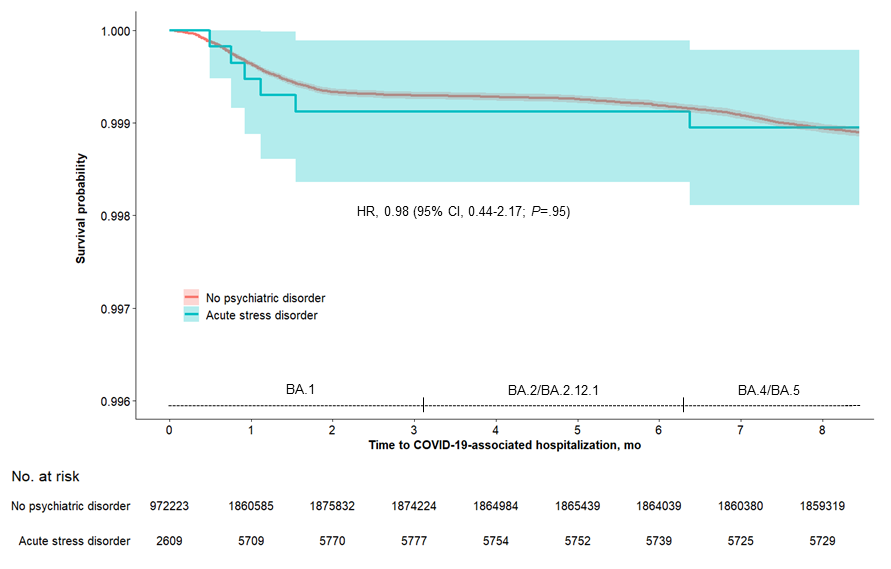


Time 0 is December 16, 2021, which was the earliest date a patient could start contributing eligible follow-up. Sites had staggered entries from December 16-26, 2021 based on the date on which the SARS-CoV-2 Omicron variant first accounted for ≥50% of all sequenced specimens at each site. Individual patients could also enter the cohort at a later date if they became eligible based on a new COVID-19 vaccination status. Periods of estimated ≥50% BA.1 sublineage predominance (as early as December 16-26, 2021), ≥50% BA.2/BA.2.12.1 sublineage predominance (as early as March 19-24, 2022), and ≥50% BA.4/BA.5 sublineage predominance (as early as June 19-29, 2022) are displayed. The shaded areas indicate 95% confidence intervals (CIs). The unadjusted hazard ratio (HR), 95% CI, and log-rank p-value that are shown were obtained from comparing patients with acute stress disorder to patients with no psychiatric disorder (reference group).

**eFigure 14. Kaplan-Meier Survival Curve of Time to COVID-19–Associated Hospitalization Comparing Adjustment Disorder with No Psychiatric Disorder.**


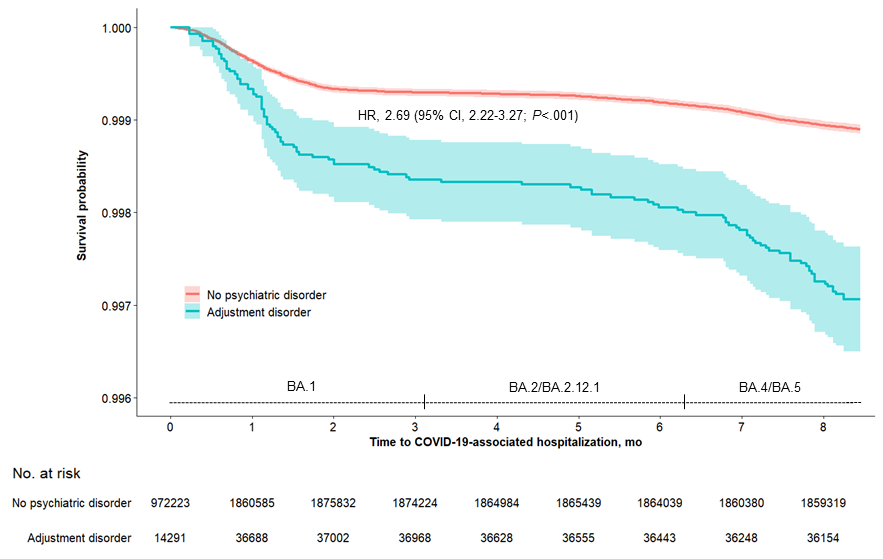


Time 0 is December 16, 2021, which was the earliest date a patient could start contributing eligible follow-up. Sites had staggered entries from December 16-26, 2021 based on the date on which the SARS-CoV-2 Omicron variant first accounted for ≥50% of all sequenced specimens at each site. Individual patients could also enter the cohort at a later date if they became eligible based on a new COVID-19 vaccination status. Periods of estimated ≥50% BA.1 sublineage predominance (as early as December 16-26, 2021), ≥50% BA.2/BA.2.12.1 sublineage predominance (as early as March 19-24, 2022), and ≥50% BA.4/BA.5 sublineage predominance (as early as June 19-29, 2022) are displayed. The shaded areas indicate 95% confidence intervals (CIs). The unadjusted hazard ratio (HR), 95% CI, and log-rank p-value that are shown were obtained from comparing patients with an adjustment disorder to patients with no psychiatric disorder (reference group).

**eFigure 15. Kaplan-Meier Survival Curve of Time to COVID-19–Associated Hospitalization Comparing Unspecified or Other Trauma-/Stressor-Related Disorder with No Psychiatric Disorder.**


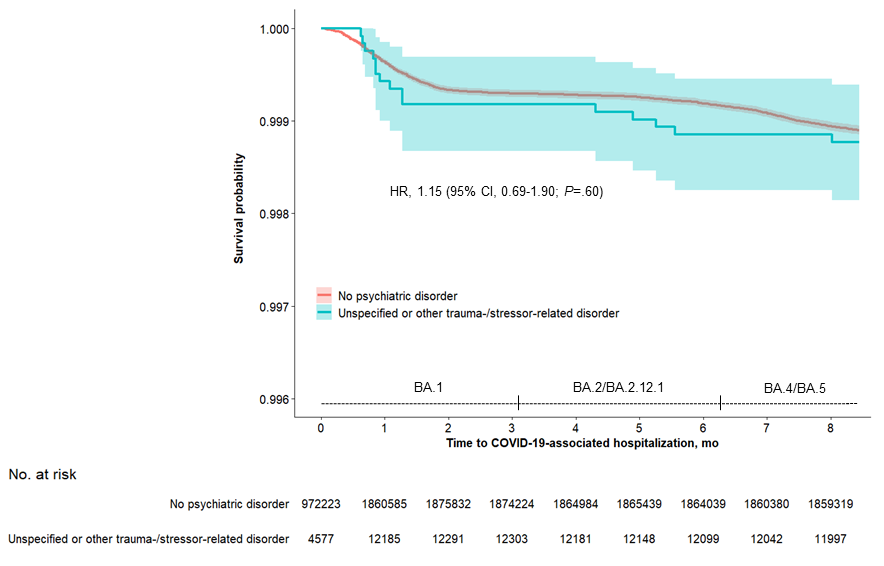


Time 0 is December 16, 2021, which was the earliest date a patient could start contributing eligible follow-up. Sites had staggered entries from December 16-26, 2021 based on the date on which the SARS-CoV-2 Omicron variant first accounted for ≥50% of all sequenced specimens at each site. Individual patients could also enter the cohort at a later date if they became eligible based on a new COVID-19 vaccination status. Periods of estimated ≥50% BA.1 sublineage predominance (as early as December 16-26, 2021), ≥50% BA.2/BA.2.12.1 sublineage predominance (as early as March 19-24, 2022), and ≥50% BA.4/BA.5 sublineage predominance (as early as June 19-29, 2022) are displayed. The shaded areas indicate 95% confidence intervals (CIs). The unadjusted hazard ratio (HR), 95% CI, and log-rank p-value that are shown were obtained from comparing patients with an unspecified or other trauma-/stressor-related disorder to patients with no psychiatric disorder (reference group).

**eFigure 16. Kaplan-Meier Survival Curve of Time to COVID-19–Associated Hospitalization Comparing Psychotic Disorder with No Psychiatric Disorder.**


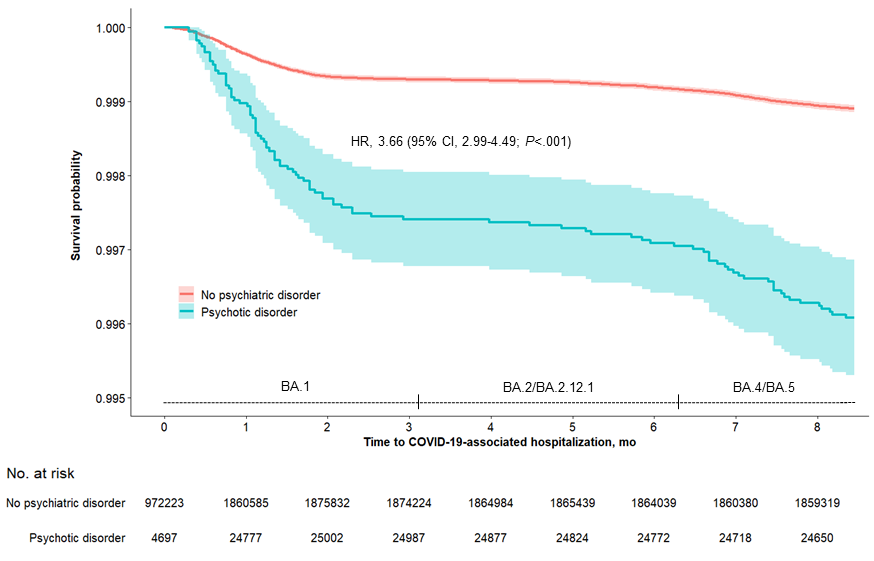


Time 0 is December 16, 2021, which was the earliest date a patient could start contributing eligible follow-up. Sites had staggered entries from December 16-26, 2021 based on the date on which the SARS-CoV-2 Omicron variant first accounted for ≥50% of all sequenced specimens at each site. Individual patients could also enter the cohort at a later date if they became eligible based on a new COVID-19 vaccination status. Periods of estimated ≥50% BA.1 sublineage predominance (as early as December 16-26, 2021), ≥50% BA.2/BA.2.12.1 sublineage predominance (as early as March 19-24, 2022), and ≥50% BA.4/BA.5 sublineage predominance (as early as June 19-29, 2022) are displayed. The shaded areas indicate 95% confidence intervals (CIs). The unadjusted hazard ratio (HR), 95% CI, and log-rank p-value that are shown were obtained from comparing patients with any mood disorder to patients with no psychiatric disorder (reference group).

**eFigure 17. Kaplan-Meier Survival Curve of Time to COVID-19–Associated Hospitalization Comparing Somatoform Disorder with No Psychiatric Disorder.**


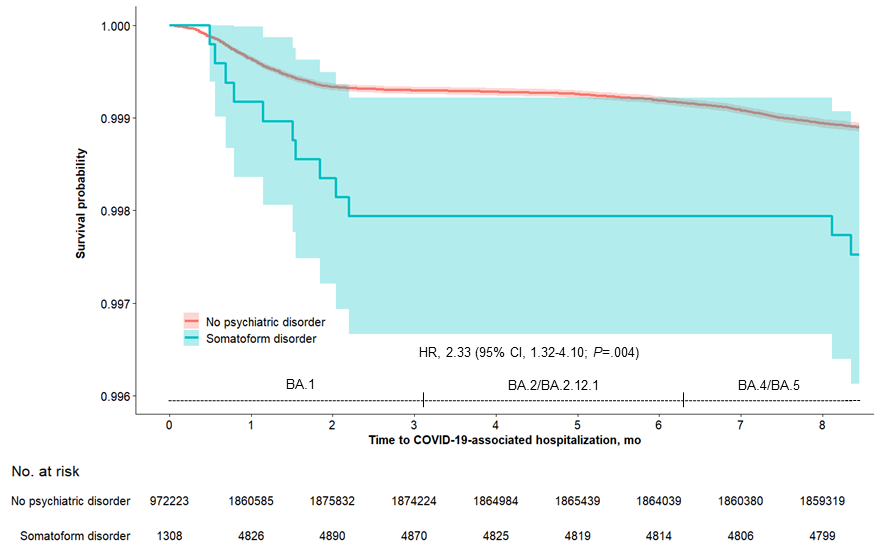


Time 0 is December 16, 2021, which was the earliest date a patient could start contributing eligible follow-up. Sites had staggered entries from December 16-26, 2021 based on the date on which the SARS-CoV-2 Omicron variant first accounted for ≥50% of all sequenced specimens at each site. Individual patients could also enter the cohort at a later date if they became eligible based on a new COVID-19 vaccination status. Periods of estimated ≥50% BA.1 sublineage predominance (as early as December 16-26, 2021), ≥50% BA.2/BA.2.12.1 sublineage predominance (as early as March 19-24, 2022), and ≥50% BA.4/BA.5 sublineage predominance (as early as June 19-29, 2022) are displayed. The shaded areas indicate 95% confidence intervals (CIs). The unadjusted hazard ratio (HR), 95% CI, and log-rank p-value that are shown were obtained from comparing patients with a somatoform disorder to patients with no psychiatric disorder (reference group).

**eFigure 18. Kaplan-Meier Survival Curve of Time to COVID-19–Associated Hospitalization Comparing Attention-Deficit Hyperactivity Disorder with No Psychiatric Disorder.**


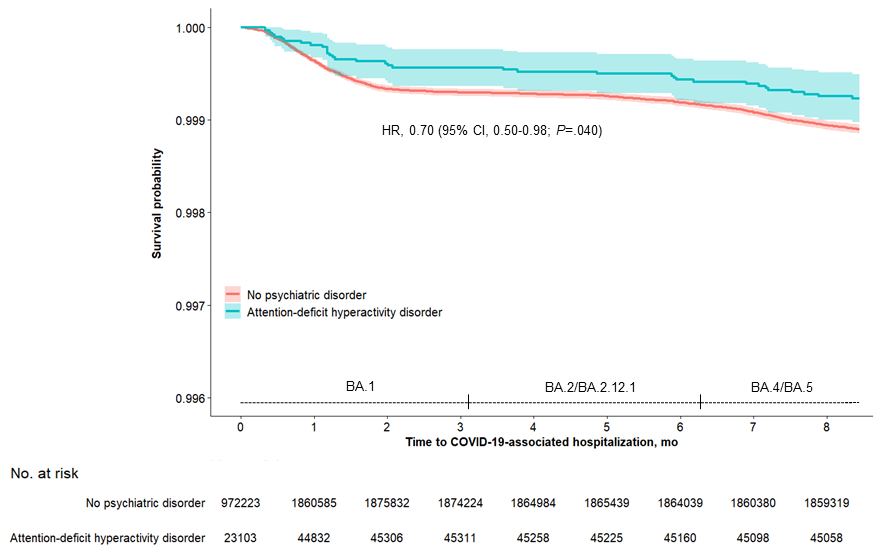


Time 0 is December 16, 2021, which was the earliest date a patient could start contributing eligible follow-up. Sites had staggered entries from December 16-26, 2021 based on the date on which the SARS-CoV-2 Omicron variant first accounted for ≥50% of all sequenced specimens at each site. Individual patients could also enter the cohort at a later date if they became eligible based on a new COVID-19 vaccination status. Periods of estimated ≥50% BA.1 sublineage predominance (as early as December 16-26, 2021), ≥50% BA.2/BA.2.12.1 sublineage predominance (as early as March 19-24, 2022), and ≥50% BA.4/BA.5 sublineage predominance (as early as June 19-29, 2022) are displayed. The shaded areas indicate 95% confidence intervals (CIs). The unadjusted hazard ratio (HR), 95% CI, and log-rank p-value that are shown were obtained from comparing patients with attention-deficit hyperactivity disorder to patients with no psychiatric disorder (reference group).

**eFigure 19. Kaplan-Meier Survival Curve of Time to COVID-19–Associated Hospitalization Comparing Eating Disorder with No Psychiatric Disorder.**


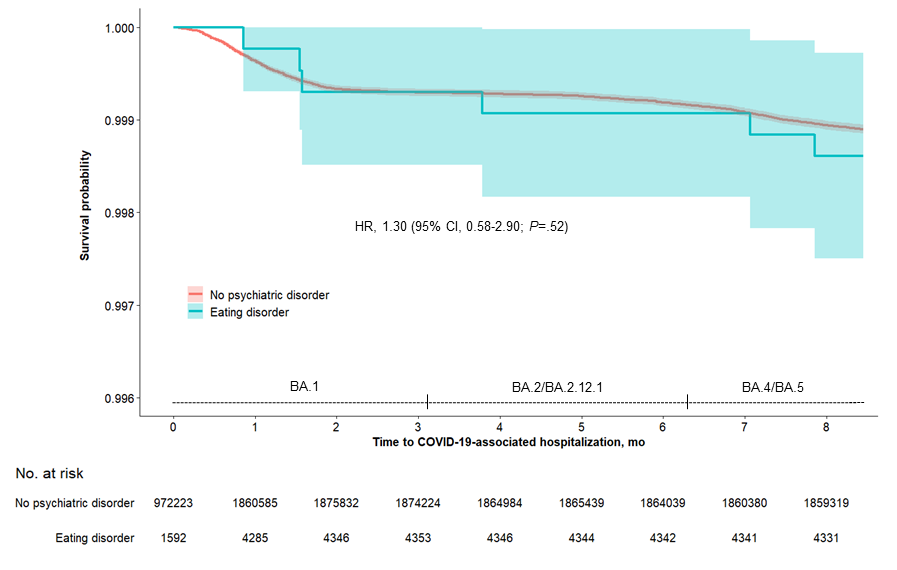


Time 0 is December 16, 2021, which was the earliest date a patient could start contributing eligible follow-up. Sites had staggered entries from December 16-26, 2021 based on the date on which the SARS-CoV-2 Omicron variant first accounted for ≥50% of all sequenced specimens at each site. Individual patients could also enter the cohort at a later date if they became eligible based on a new COVID-19 vaccination status. Periods of estimated ≥50% BA.1 sublineage predominance (as early as December 16-26, 2021), ≥50% BA.2/BA.2.12.1 sublineage predominance (as early as March 19-24, 2022), and ≥50% BA.4/BA.5 sublineage predominance (as early as June 19-29, 2022) are displayed. The shaded areas indicate 95% confidence intervals (CIs). The unadjusted hazard ratio (HR), 95% CI, and log-rank p-value that are shown were obtained from comparing patients with an eating disorder to patients with no psychiatric disorder (reference group).

**eFigure 20. Kaplan-Meier Survival Curve of Time to COVID-19–Associated Hospitalization Comparing Personality Disorder with No Psychiatric Disorder.**


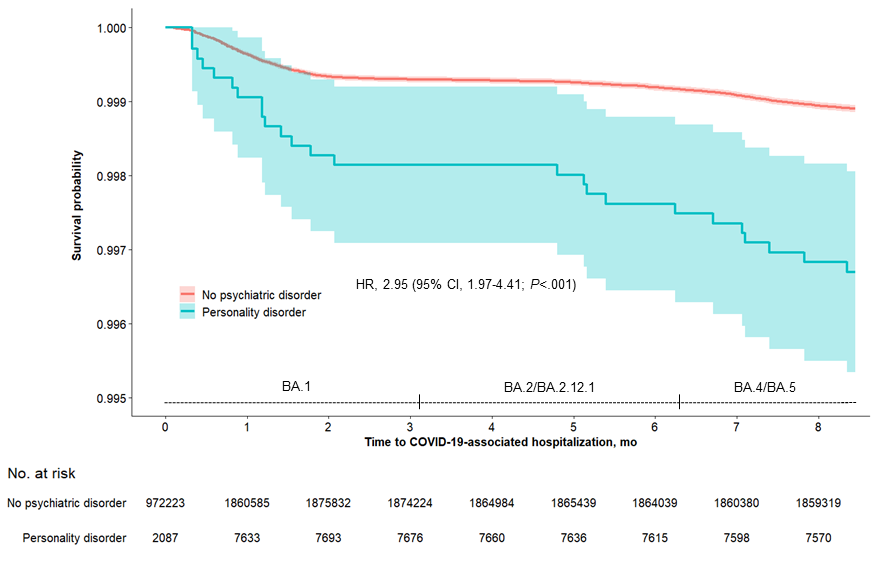


Time 0 is December 16, 2021, which was the earliest date a patient could start contributing eligible follow-up. Sites had staggered entries from December 16-26, 2021 based on the date on which the SARS-CoV-2 Omicron variant first accounted for ≥50% of all sequenced specimens at each site. Individual patients could also enter the cohort at a later date if they became eligible based on a new COVID-19 vaccination status. Periods of estimated ≥50% BA.1 sublineage predominance (as early as December 16-26, 2021), ≥50% BA.2/BA.2.12.1 sublineage predominance (as early as March 19-24, 2022), and ≥50% BA.4/BA.5 sublineage predominance (as early as June 19-29, 2022) are displayed. The shaded areas indicate 95% confidence intervals (CIs). The unadjusted hazard ratio (HR), 95% CI, and log-rank p-value that are shown were obtained from comparing patients with a personality disorder to patients with no psychiatric disorder (reference group).

**eFigure 21. Kaplan-Meier Survival Curve of Time to COVID-19–Associated Hospitalization Comparing Dissociative or Conversion Disorder with No Psychiatric Disorder.**


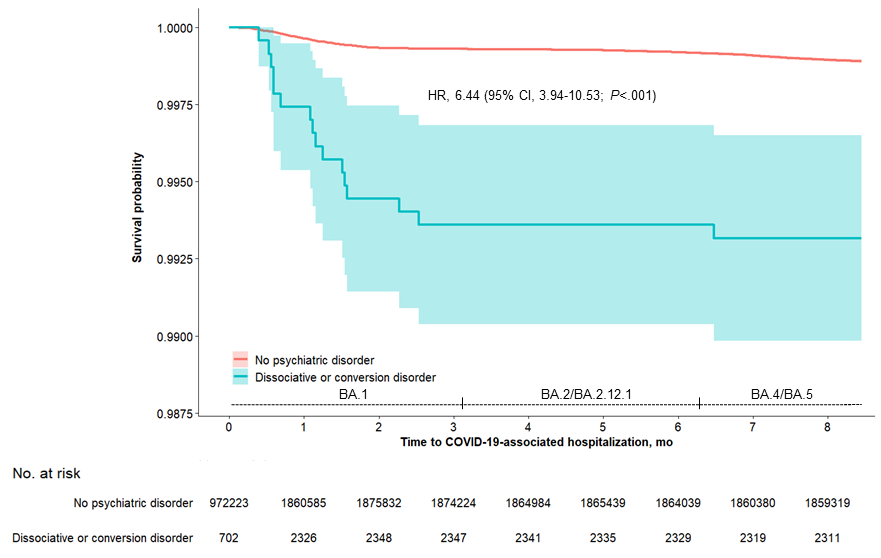


Time 0 is December 16, 2021, which was the earliest date a patient could start contributing eligible follow-up. Sites had staggered entries from December 16-26, 2021 based on the date on which the SARS-CoV-2 Omicron variant first accounted for ≥50% of all sequenced specimens at each site. Individual patients could also enter the cohort at a later date if they became eligible based on a new COVID-19 vaccination status. Periods of estimated ≥50% BA.1 sublineage predominance (as early as December 16-26, 2021), ≥50% BA.2/BA.2.12.1 sublineage predominance (as early as March 19-24, 2022), and ≥50% BA.4/BA.5 sublineage predominance (as early as June 19-29, 2022) are displayed. The shaded areas indicate 95% confidence intervals (CIs). The unadjusted hazard ratio (HR), 95% CI, and log-rank p-value that are shown were obtained from comparing patients with a dissociative or conversion disorder to patients with no psychiatric disorder (reference group).

**eFigure 22. Associations Between Number or Combination of Psychiatric Disorder Types and COVID-19–Associated Hospitalization.**

**
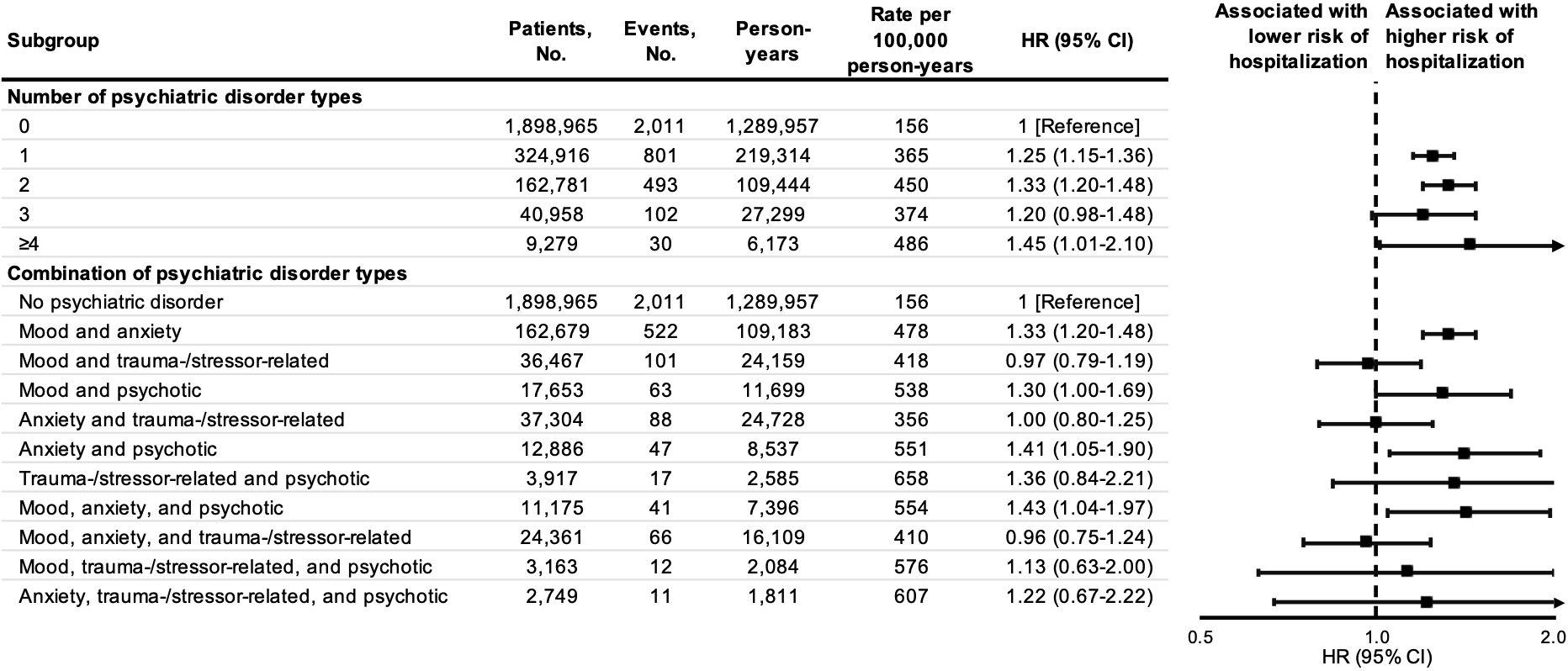
**

A hazard ratio (HR) >1.0 indicates that the respective number or combination of psychiatric disorders was associated with a higher risk of COVID-19–associated hospitalization. For combinations of psychiatric disorders, each HR was obtained from a separate model. HRs were adjusted for site, age (natural spline with 4 knots), sex (male, female, unknown), race and ethnicity (Asian, Black, Hispanic, white, other, unknown), Medicaid coverage (yes, no, unknown), underlying respiratory condition (yes, no), underlying non-respiratory condition (yes, no), number of underlying medical conditions (square-root transformed), number of SARS-CoV-2 test records documented in the patient's electronic medical record prior to the start of the study period (0, 1, 2-4, ≥5), and time-varying mRNA COVID-19 vaccination status (unvaccinated, two doses 14-149 days earlier, two doses ≥150 days earlier, three doses 7-119 days earlier, three doses ≥120 days earlier, four doses 7-59 days earlier, four doses ≥60 days earlier). CI indicates confidence interval.

**eFigure 23. Associations of Any Psychiatric Disorder and Other Underlying Medical Conditions with COVID-19–Associated Hospitalization.**

**
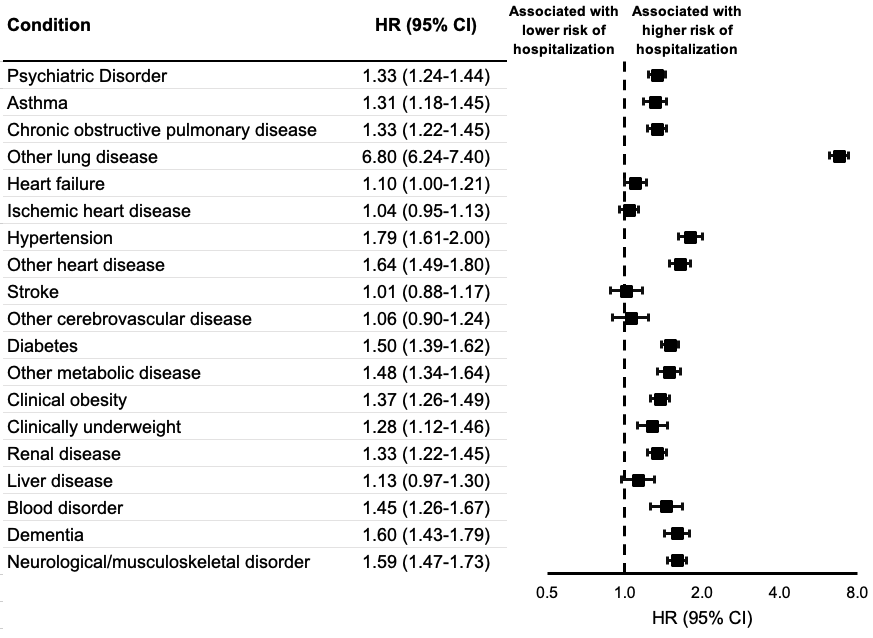
**

A hazard ratio (HR) >1.0 indicates that the respective condition was associated with a higher risk of COVID-19–associated hospitalization. All HRs were obtained from the same model. HRs were adjusted for site, age (natural spline with 4 knots), sex (male, female, unknown), race and ethnicity (Asian, Black, Hispanic, white, other, unknown), Medicaid coverage (yes, no, unknown), number of SARS-CoV-2 test records documented in the patient's electronic medical record prior to the start of the study period (0, 1, 2-4, ≥5), time-varying mRNA COVID-19 vaccination status (unvaccinated, two doses 14-149 days earlier, two doses ≥150 days earlier, three doses 7-119 days earlier, three doses ≥120 days earlier, four doses 7-59 days earlier, four doses ≥60 days earlier), and each condition listed. CI indicates confidence interval.

**eFigure 24. Associations Between Vaccination Status and COVID-19–Associated Hospitalization, Stratified by Psychiatric Disorder Type.**


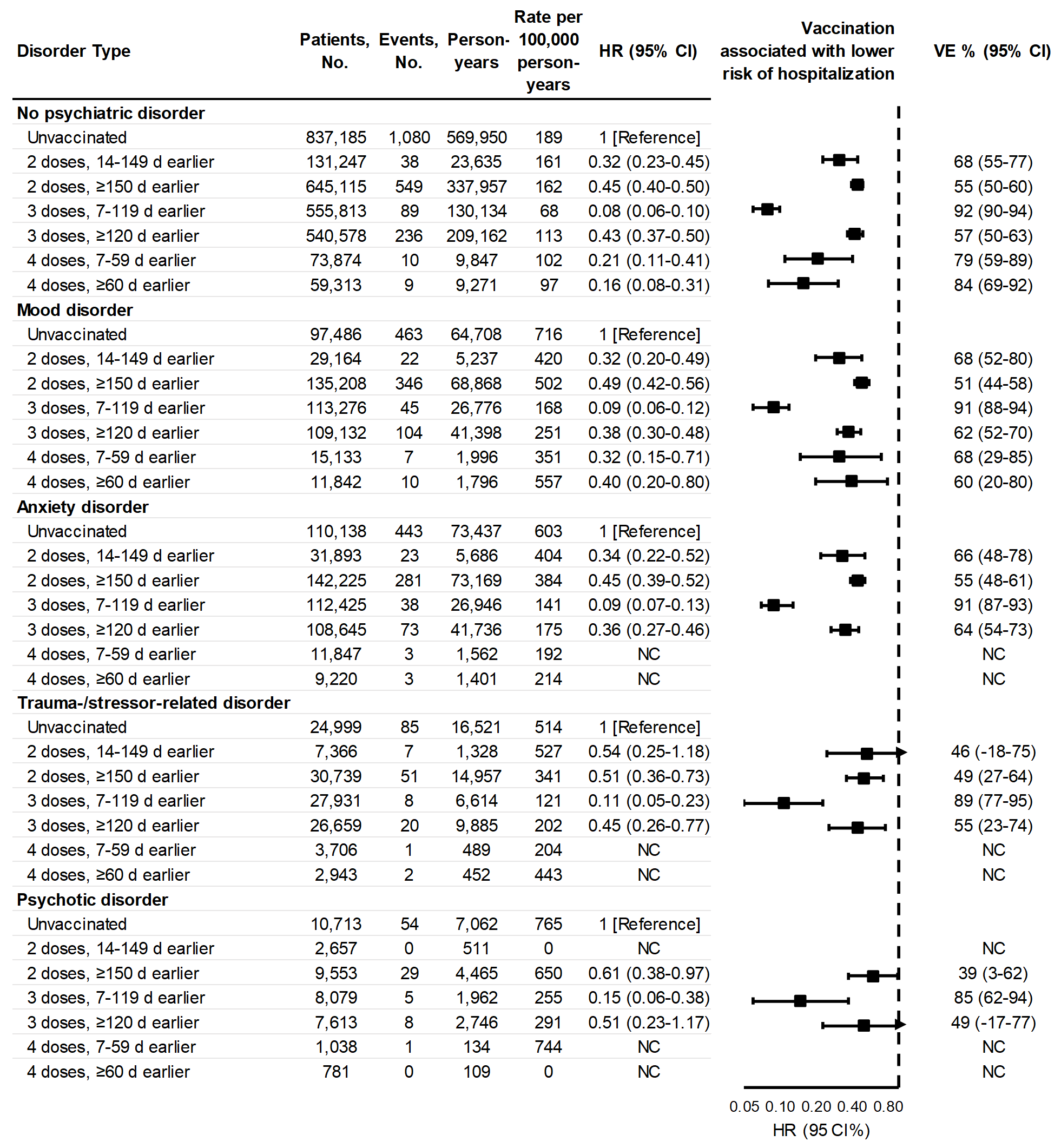


A hazard ratio (HR) <1.0 indicates that being vaccinated versus unvaccinated was associated with a lower risk of COVID-19–associated hospitalization. HRs were adjusted for site, age (natural spline with 4 knots), sex (male, female, unknown), race and ethnicity (Asian, Black, Hispanic, white, other, unknown), Medicaid coverage (yes, no, unknown), underlying respiratory condition (yes, no), underlying non-respiratory condition (yes, no), number of underlying medical conditions (square-root transformed), and number of SARS-CoV-2 test records documented in the patient's electronic medical record prior to the start of the study period (0, 1, 2-4, ≥5). For four doses 7-59 days earlier, only person-time after April 5, 2022 among patients aged ≥50 years was analyzed. For four doses ≥60 days earlier, only person-time after May 28, 2022 among patients aged ≥50 years was analyzed. HRs were not calculated (NC) for subgroups with both fewer than five events and less than 2,000 total person-years. Vaccine effectiveness (VE) for prevention of COVID-19–associated hospitalization was estimated from HRs using the equation: VE = (1-HR) x 100%. CI indicates confidence interval.

**eFigure 25. Association Between COVID-19–Associated Hospitalization with Prior Vaccination, Stratified By Age Group and Psychiatric Disorder Status, Using a Case-Control Test-Negative Design.**


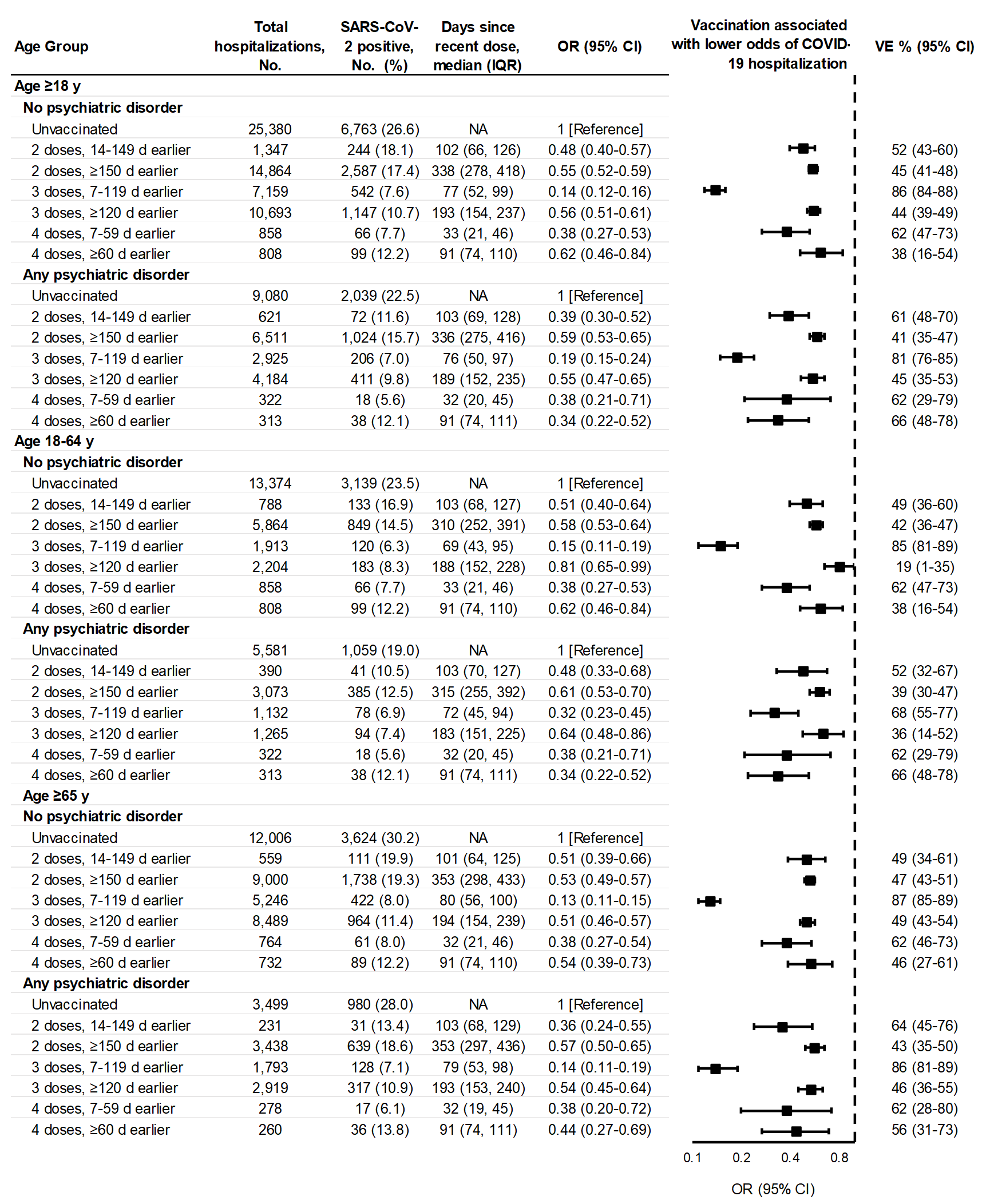


An odds ratio (OR) <1.0 indicates that being vaccinated versus unvaccinated was associated with a lower odds of COVID-19–associated hospitalization. ORs were adjusted for age, geographic region, calendar time (days since January 1, 2021), and local virus circulation (percentage of SARS-CoV-2–positive results from testing within the counties surrounding the facility on the date of the encounter) and weighted for inverse propensity to be vaccinated or unvaccinated (calculated separately for each OR estimate). Generalized boosted regression trees were used to estimate the propensity to be vaccinated based on the following demographic, hospital, and medical factors: age, sex, race, ethnicity, Medicaid status, calendar date, geographic region, local SARS-CoV-2 circulation on the day of each medical visit, urban-rural classification of facility, hospital type, number of hospital beds, chronic respiratory condition, chronic non-respiratory condition, asthma, chronic obstructive pulmonary disease, other chronic lung disease, heart failure, ischemic heart disease, hypertension, other heart disease, stroke, other cerebrovascular disease, diabetes type 1, diabetes type 2, diabetes due to underlying conditions or other specified diabetes, other metabolic disease (excluding diabetes), clinical obesity, clinical underweight, renal disease, liver disease, blood disorder, dementia, other neurological/musculoskeletal disorder, Down syndrome, and the presence of at least one prior molecular or rapid antigen SARS-CoV-2 test record documented in the electronic medical record ≥15 days before the medical encounter date (pre-vaccination, if vaccinated). For four doses 7-59 days earlier, only hospitalizations on/after April 5, 2022 among patients aged ≥50 years were analyzed. For four doses ≥60 days earlier, only hospitalizations on/after May 28, 2022 among patients aged ≥50 years were analyzed. Vaccine effectiveness (VE) for prevention of COVID-19–associated hospitalization was estimated from the adjusted ORs presented in this table with the equation: VE = (1-adjusted OR) x 100%. CI indicates confidence interval; IQR, interquartile range; NA, not applicable.
